# Comprehensive evaluation of clinical phenotypes and pathogenic features in late-onset monogenic inflammatory bowel disease: a comparative study with infantile-onset cases

**DOI:** 10.1186/s12876-025-04041-4

**Published:** 2025-06-05

**Authors:** Haoying Liu, Yinxian Shen, Jiaxin Xu, Shangzhan Huang, TingTing Qin, Qi Zhou, Jiazhi Liao, Fang Xiao

**Affiliations:** 1 Department of Gastroenterology, Tongji Hospital, Tongji Medical College, Huazhong University of Science and Technology, Wuhan, China; 2 Department of Biliary-Pancreatic Surgery, Tongji Hospital of Tongji Medical College, Huazhong University of Science and Technology, Wuhan, China

**Keywords:** IBD, Monogenic disorders, Late-onset, Phenotypes

## Abstract

**Background:**

Monogenic inflammatory bowel disease (mIBD) in patients with onset after the age of 16 has been increasingly recognized, but these reports are sporadic and lack a systematic overview, leaving the clinical and genetic characteristics poorly understood. This study aimed to characterize these late-onset mIBD (LO-mIBD), using an infantile-onset population as a control.

**Methods:**

Data were extracted from eligible case reports, case series, and cohorts published between January 1990 and October 2023. A comprehensive search of PubMed and Web of Science was conducted following PRISMA guidelines. Demographic, genetic, phenotypic, and genotypic data were collected for the comparative analysis between LO-mIBD (> 16 years) and infantile-onset mIBD (IO-mIBD, < 2 years) cases that met the inclusion criteria.

**Results:**

A total of 436 IO-mIBD and 110 LO-mIBD cases were included in the analysis. Patients with LO-mIBD had significantly lower rates of parental consanguinity and a higher frequency of heterozygous mutations in autosomal dominant genes compared with IO-mIBD cases. Meanwhile, patients with LO-mIBD more commonly presented with Crohn’s disease (CD)-like gastrointestinal phenotypes. They exhibited markedly higher rates of intestinal ulcers, strictures, small intestinal involvement, and granulomatous pathology but fewer instances of villous atrophy, perianal disease, oral lesions, recurrent infections, and fever compared with IO-mIBD cases. The genes most commonly implicated in LO-mIBD were *SLCO2A1*, *GUCY2C*, *CTLA4*, *CYBB*, and *SLC26A3*. Defects affecting intestinal epithelial function and phagocytic activity were more prominent in LO-mIBD than in IO-mIBD.

**Conclusions:**

Patients with LO-mIBD exhibited diverse intestinal phenotypes dominated by CD-like disease, with gene defects predominantly involving epithelial and phagocytic functions. Genetic counseling, genotype-phenotype mapping, and functional validation of key pathways are critical for further improving the clinical management of patients with LO-mIBD.

**Supplementary Information:**

The online version contains supplementary material available at 10.1186/s12876-025-04041-4.

## Introduction

Monogenic inflammatory bowel disease (mIBD) refers to a rare condition characterized by clinical features similar to those of inflammatory bowel disease (IBD), caused by pathogenic mutations in a single gene [1, 2]. These monogenic mutations can lead to various immune or intestinal epithelial dysfunctions, ultimately resulting in intestinal inflammation [3]. Unlike the more prevalent polygenic IBD, such as Crohn’s disease (CD) and ulcerative colitis (UC), mIBD typically follows a more distinct pattern of genetic inheritance that can be diagnosed through genetic sequencing [4, 5]. Additionally, patients with mIBD exhibit various degrees of penetrance and diverse phenotypes in the gastrointestinal (GI) tract, depending on the specific genes involved [6]. For example, patients with interleukin-10 (IL-10) signaling deficiency (mutations in *IL10*, *IL10RA*, or *IL10RB*) present with very early-onset severe intestinal and perianal disease [7], whereas those with *FOXP3* deficiency exhibit multisystem autoimmune symptoms and nonspecific enteropathy with villous atrophy [8]. As attention to mIBD grows, the number of reported mIBD cases has considerably increased, as has the discovery of new pathogenic genes [1, 9]. A total of 102 pathogenic genes implicated in mIBD have been identified recently, most of which are associated with primary immunodeficiency disorders (PIDs) or inborn errors of immunity (IEIs) [6, 10].

The GI manifestations of monogenic IBD usually appear at an early age, with patients often classified as the A1 type under the Montreal classification of IBD (before 16 years of age) [11], primarily during infancy or early childhood [9, 12]. The earlier onset of symptoms is one of the key features of mIBD, differentiating mIBD from classic IBD, which commonly emerges during adolescence or early adulthood [13, 14]. However, several patients with monogenic disorders develop IBD-like symptoms beyond childhood [15]. With advances in genetic technology and increased attention to mIBD, many adolescents diagnosed with IBD (Montreal Classification A2/A3, > 16 years) were later identified as having mIBD, especially in cases with atypical features or those refractory to treatment [16–18]. These instances of late-onset mIBD (LO-mIBD) underscore that mIBD is not exclusive to infants, and adult gastroenterologists may encounter such patients, indicating the need to understand the characteristics of the late-onset population. However, LO-mIBD has only been documented in isolated cases (e.g., published late-onset cases with SLCO2A1 [18], SLC26A3 [19], and BTK [20] deficiencies), and to our knowledge, no studies have systematically characterized its clinical and pathophysiological characteristics.

Collectively, LO-mIBD, particularly in individuals with onset after 16 years of age, has historically been overlooked but is closely relevant to adult gastroenterology. To address this gap, we systematically evaluated the clinical features, genotypic characteristics, and pathophysiological mechanisms of LO-mIBD by integrating globally reported cases. In this study, LO-mIBD refers to individuals with mIBD who developed IBD-like phenotypes after the age of 16, corresponding to the A2/A3 type as defined by the Montreal classification [11]. This subgroup was designed as an initial step toward characterizing this understudied population and to ensure clinical translatability in adult gastroenterology practice. In addition, despite existing gaps in LO-mIBD research, the features of infantile-onset mIBD (IO-mIBD, < 2 years) populations have been increasingly elucidated in recent studies [21–23]. Therefore, we conducted a comparative analysis of LO-mIBD using IO-mIBD cases as a control. These efforts are critical for providing initial guidance for adult gastroenterology practices and valuable insights for future research on LO-mIBD.

## Methods

### Study design and data collection

According to previous studies [6, 9], monogenic IBD was defined as cases with confirmed monogenic disease and IBD-like GI phenotypes. The age at onset of IBD-like manifestations was assessed as the time of endoscopic confirmation or the time at which intestinal symptoms appeared if there was a delay of more than 1 year before endoscopy, as described by Bolton et al. [6].

**Fig. 1:**
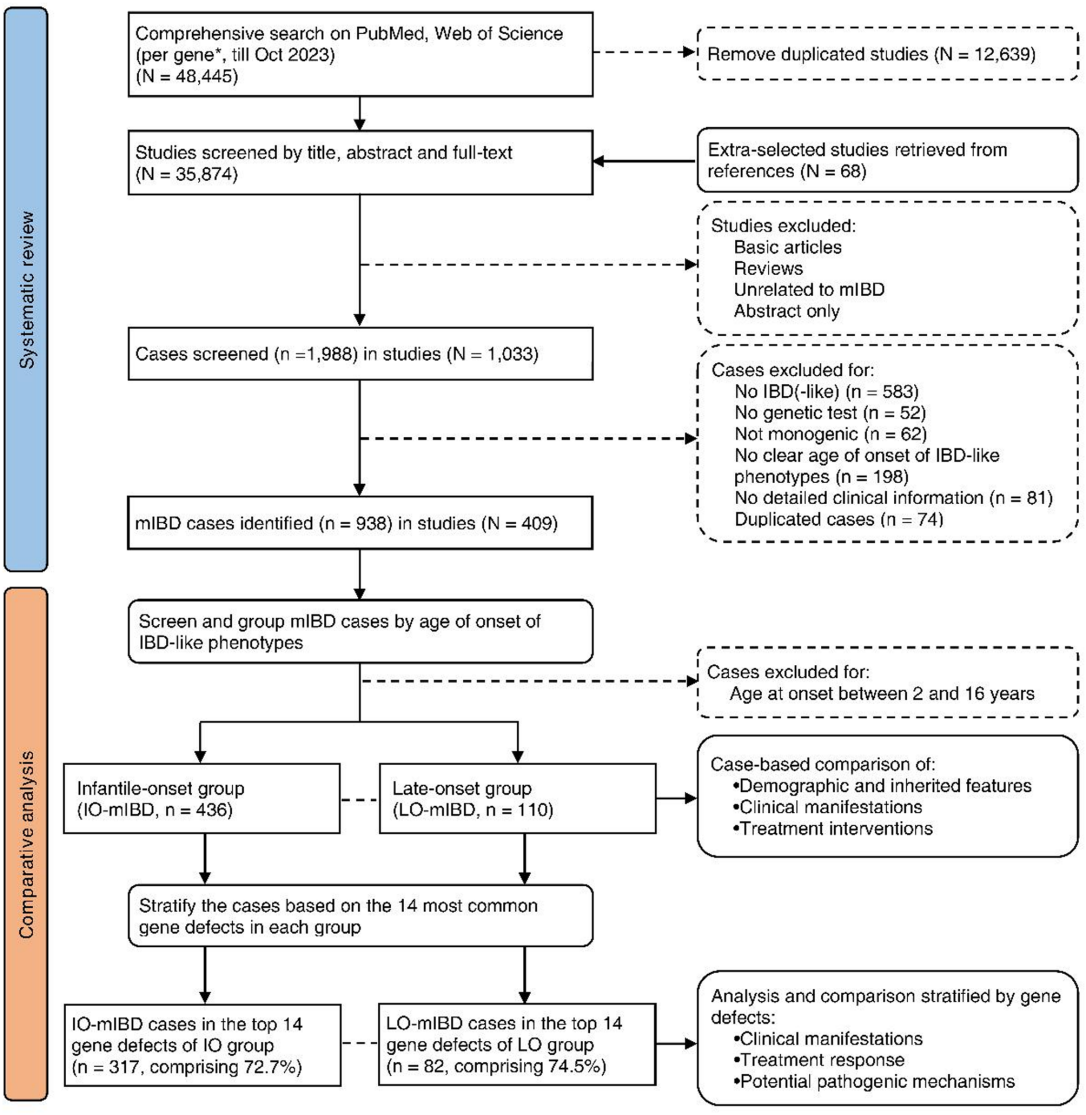
PRISMA flow chart of the study design

A comprehensive search was conducted for articles published between January 1990 and October 2023 using PubMed and Web of Science, according to the Preferred Reporting Items for Systematic Reviews and Meta-Analyses (PRISMA) guidelines (Fig. 1). The search terms included “IBD”, “inflammatory bowel disease”, “Crohn’s”, “enterocolitis”, and “enteropathy”, combined with the names or abbreviations of genes, proteins, monogenic diseases, or syndromes. A search was performed for each of the 102 defined mIBD genes [2, 6]. Google Scholar, the related article function, and reference lists were also used to supplement the published studies; no language restrictions were made. The titles, abstracts, and full texts were evaluated to exclude basic scientific articles and reviews without clinical cases, articles unrelated to mIBD, conference abstracts, and duplicate articles. Case details were further scrutinized to screen for IO-mIBD and LO-mIBD during the full-text evaluation of the eligible articles. The inclusion criteria for the cases were as follows: (1) definite diagnosis of monogenic disease and corresponding pathogenic single-gene mutations confirmed by genetic sequencing, including Sanger sequencing, targeted gene panel sequencing (TGPS), targeted next-generation sequencing (TNGS), whole-exome sequencing (WES), and whole-genome sequencing (WGS); (2) presentation of IBD-like GI phenotypes or chronic intestinal inflammation based on endoscopic or histological findings, including suspected diagnoses of IBD, intestinal Behçet’s disease, granulomatous colitis, autoimmune enteropathy, chronic nonspecific enterocolitis, cryptogenic multifocal ulcerous stenosing enteritis (CMUSE), chronic non-specific multiple ulcers of the small intestine (CNSU), and common variable immunodeficiency-associated enteropathy; (3) the age at onset of IBD-like manifestations was either younger than 2 years or older than 16 years; and (4) detailed clinical information for individual cases, including at least two of the following four aspects: demographic information, GI findings, extraintestinal manifestations (EIMs), and treatment information. The exclusion criteria were as follows: (1) no genetic testing results or no genetic testing performed; (2) mutations in multiple genes; (3) no evidence of intestinal inflammation, or enterocolitis caused by other known factors, such as pathogens; (4) IBD-like manifestations that appeared between 2 and 16 years of age; and (5) insufficient details of individual cases in cohort studies or case series.

The following data were collected for each case: pathogenic genes, mutation zygosity, age at onset of IBD-like disease, sex, parental consanguinity, family history, GI phenotypes labeled by the authors, GI symptoms and complications, lesion sites, endoscopic and pathological features, EIMs and comorbidities, and treatment interventions and responses. See the Supporting Information (Supplementary Table 1) for the detailed classification and definition of each feature. Briefly, the mutation zygosities included homozygotes, compound heterozygotes, heterozygotes, and hemizygotes. GI phenotypes were classified into five categories: CD, UC, IBD-unclassified (IBDU) or indeterminate colitis (IC), IBD (IBD cases without delineating subtypes), and others (other chronic intestinal inflammatory diseases resembling IBD). Intestinal pathological features were classified into five patterns, as described by Wilkins et al. [24]. EIMs are assessed based on the systemic manifestations associated with IBD [25], along with other specific comorbidities, such as recurrent infections, fever, malignancy, etc. Treatment interventions include traditional options for classic IBD and several other special approaches for mIBD, such as hematopoietic stem cell transplantation (HSCT). Response to therapy refers to symptomatic or endoscopic remission of the GI tract, excluding transient relief. The variants in the included cases were re-examined using the classification tool “Franklin” (https://franklin.genoox.com/clinical-db/home) based on the latest ACMG guidelines [26]. The quality of the included cases was assessed using the Joanna Briggs Institute (JBI) critical appraisal checklists for case reports and case series (Supplementary Table 2A, B). Each item was scored as 1 (“yes”) or 0 (“no/unclear”), with maximum total scores of 8 and 10, respectively. Studies scoring > 7, 4–6, and 0–3 were considered high, moderate, and low quality, respectively. Two authors independently evaluated the procedure.

We comparatively analyzed the demographic, inherited, and clinical data of the patients with LO-mIBD and IO-mIBD. The 14 most common genes were used for further stratification to investigate the genotype-associated phenotypic features and treatment effectiveness in those with LO-mIBD and IO-mIBD. Finally, the immune process was evaluated according to the latest IEIs/PIDs classification [10], and Gene Ontology (GO) enrichment was analyzed using Metascape [27] to compare the functional pathways and underlying pathophysiological mechanisms of the most common genes in each group.

### Statistical analysis

Median and interquartile range (IQR) were used to describe the age at onset, whereas numbers and percentages were used for other categorical variables. The chi-square test or Fisher’s exact test was used to compare rates or proportions, and the Bonferroni method was used for p-value adjustment in post hoc multiple comparisons. Univariate binary logistic regression was used to analyze the correlation between variant zygosity and LO-mIBD, and multifactorial logistic regression was used to adjust potential confounders, including sex, consanguineous history, GI complications, and genotype. The results are presented as the odds ratio (OR) of LO-mIBD relative to IO-mIBD, along with its 95% confidence interval (CI). Sensitivity analysis was conducted to evaluate the potential risk of bias arising from missing data, assuming an RI_LTFU/FU_ of 0.5 for the group with the higher incidence and 1 for the group with the lower incidence [28]. P values less than 0.05 were considered statistically significant. All statistical analyses were conducted using the SPSS software (v25.0; IBM, New York, USA), and figures were generated using GraphPad Prism (v10.1) and Origin (v2025).

## Results

### Demographic and inherited features of patients with late-onset and infantile-onset mIBD

We initially screened 1,988 cases from 1,033 eligible articles by searching for 102 defined mIBD genes. According to our criteria, a total of 938 mIBD cases involving 88 of 102 genes were discovered, among which 546 cases involving 77 genes were ultimately included in the comparative analysis (Fig. 1).

Specifically, 436 cases of IO-mIBD and 110 cases of LO-mIBD were included, with males accounting for 61.7% and 48.1% of the cases, respectively (*p* = 0.011). The median age at the onset of the IBD-like disease was 0.16 years (IQR: 0.04–0.50) in patients with IO-mIBD and 24.00 years (IQR: 18.00–35.25) in those with LO-mIBD. Asians comprised the largest proportion of included cases (36.45%), followed by European/Caucasians (12.45%), with race/ethnicity unreported in 34.98% of cases (Supplementary Fig. 3b). Parental consanguinity, referring to the offspring of consanguineous parents, was less prevalent in patients with LO-mIBD than in those with IO-mIBD (*p* = 0.015, OR: 0.49, 95% CI: 0.27–0.88), and it was mainly found in patients with homozygous mutations (100% in LO-mIBD and 98.1% in IO-mIBD). No significant differences in the family history of either monogenic disorders or IBD-like manifestations were observed between the groups (*p* > 0.05).

Homozygosity was the most common type of zygosity in both groups. However, patients with LO-mIBD had a larger proportion of heterozygotes and a lower proportion of compound heterozygotes compared with IO-mIBD cases (Bonferroni-adjusted *p* < 0.05, Table 1). Moreover, the results of binary logistic regression analysis revealed a significant correlation between the zygosity type of heterozygosity and LO-mIBD (*p* = 0.001, OR: 2.56, 95% CI: 1.45–4.52, using homozygotes as the reference, Supplementary Table 3). Multifactorial regression analyses adjusted for genotype, rather than sex or consanguineous history, significantly altered the differences in zygosity, suggesting that genotype is a key factor influencing the differences in zygosity between the groups (Supplementary Table 4). Further analysis showed that the proportion of patients with autosomal dominant gene defects was also higher in the LO-mIBD group (23.6% vs. 10.8%, adjusted *p* < 0.05), with *GUCY2C*, *CTLA4*, and *SYK* deficiencies being the most common.

A total of 65 of the 77 pathogenic genes were reported in patients with IO-mIBD, 36 in patients with LO-mIBD, and 24 overlapped between the groups (Supplementary Fig. 1). The median age of onset of IBD-like phenotypes varied across different genes, ranging from 0.02 to 27 years. Specifically, 33 genes had a median age at onset of less than 2 years, whereas 9 had an onset age greater than 16 years, including *CARD8*, *PIK3R1*, *ICOS*, *PI4KA*, *TRNT1*, *TYMP*, *IRF2BP2*, *GUCY2C*, and *SYK* (Supplementary Fig. 2).

The comprehensive search and screening are based on PRISMA guidelines. A total of 436 cases of infantile-onset mIBD (IO-mIBD) and 110 cases of late-onset mIBD (LO-mIBD) meeting the criteria were included in the comparative analysis. The cases were then stratified according to the top 14 most common gene defects in each group to analyze the clinical and pathogenic characteristics associated with these defects.

^*^. A total of 102 mIBD genes identified by Bolton et al. [6] and Kammermeier et al. [2].

**Table 1:** Comparison of demographic and inherited features in patients with late-onset vs. infantile-onset mIBD

	LO-mIBD	IO-mIBD	OR (95% CI)	*P* value
	% (*n*/*N*^†^)	% (*n*/*N*^†^)		
**Total number of cases**	110	436		
**Onset age of IBD** (years) (median, IQR)	24.00 (18.00, 35.25)	0.16 (0.04, 0.50)		
**Sex (male)**	48.1 (52/108)	61.7 (265/428)	0.57 (0.38, 0.88)	0.011
**Parental consanguinity**	17.4 (16/92)	30.1 (107/356)	0.49 (0.27, 0.88)	0.015
**Family history of similar monogenic disorders**	45.7 (42/92)	36.5 (130/356)	1.46 (0.92, 2.32)	0.108
**Family history of IBD-like manifestations**	28.3 (26/92)	31.5 (112/356)	0.86 (0.52, 1.42)	0.553
**Zygosity**				< 0.001
Homozygotes	40.9 (45/110)	42.0 (183/436)	(Ref)	(Ref)
Compound heterozygotes	19.1 (21/110) ^**^	28.7 (125/436)	0.68 (0.39, 1.20)	0.187
Heterozygotes	26.4 (29/110) ^**^	10.6 (46/436)	2.56 (1.45, 4.52)	0.001
Hemizygotes	13.6 (15/110)	18.8 (82/436)	0.74 (0.392, 1.41)	0.365

IQR: interquartile range

^†^. n = number of cases presenting the corresponding features; N = number of cases with available information about the features in the reports

^**^. Bonferroni-adjusted *P* < 0.05 between LO-mIBD and IO-mIBD

### Clinical manifestations of late-onset mIBD compared with infantile-onset mIBD

Among the five categories of GI phenotypes analyzed, the CD-like phenotype was the most common in LO-mIBD (43.6%), followed by other intestinal inflammatory diseases, particularly chronic nonspecific multiple ulcers of the small intestine (CNSU) and chronic enteropathy associated with the *SLCO2A1* gene (CEAS) (17.3%). The prevalence of these GI phenotypes in the LO-mIBD group was significantly higher than that in the IO-mIBD group (adjusted *p* < 0.05, Table 2), which was more commonly labeled as IBD without delineating subtypes (adjusted *p* < 0.05).

Patients with LO-mIBD presented significantly more incidences of abdominal pain (*p* < 0.001, OR: 4.62, 95% CI: 2.80–7.62) but fewer diarrhea, hematochezia, and perianal diseases compared with IO-mIBD cases (*p* < 0.001, OR: 0.16, 95% CI: 0.09–0.29; OR: 0.29, 95% CI: 0.18–0.48; OR: 0.10, 95% CI: 0.05–0.20, respectively). GI complication of stricture was commonly reported in patients with LO-mIBD (31.5%), markedly higher than in those with IO-mIBD (*p* = 0.002, OR: 2.24, 95% CI: 1.32–3.81). Complications such as intestinal fistula and perforation were similar between the groups (*p* > 0.05). The colon was the most common site of lesions in both groups. However, small intestinal lesions were relatively more common in patients with LO-mIBD than in those with IO-mIBD (*p* < 0.001, OR: 3.37, 95% CI: 2.08–5.45), while colonic (*p* < 0.001, OR: 0.25, 95% CI: 0.15–0.43) and rectal lesions (*p* = 0.021, OR: 0.45, 95% CI: 0.22–0.90) showed the opposite. Patients with LO-mIBD also had higher incidences of intestinal ulcers (*p* = 0.008, OR: 3.21, 95% CI: 1.31–7.86) but fewer pseudopolyps (*p* = 0.006, OR: 0.21, 95% CI: 0.06–0.70) and villous atrophy (*p* = 0.005, OR: 0.20, 95% CI: 0.06–0.68) compared with IO-mIBD cases. Among the five pathological patterns, the classic pattern—chronic active enteritis—was most prevalent in both groups. The lymphocytic pattern, characterized by lymphocytosis and nodular lymphoid hyperplasia, as well as the granulomatous pattern, were significantly more common in patients with LO-mIBD than in those with IO-mIBD (*p* = 0.024, OR: 2.36, 95% CI: 1.10–5.05; *p* = 0.025, OR: 2.43, 95% CI: 1.10–5.38, respectively).

Table 3 shows the EIMs and comorbidities reported by patients with LO-mIBD and IO-mIBD. Notably, those with LO-mIBD had significantly higher instances of anemia and musculoskeletal abnormalities than those with IO-mIBD (*p* = 0.004, OR: 1.98, 95% CI: 1.23–3.18; *p* < 0.001, OR: 3.45, 95% CI: 2.06–5.77, respectively), whereas growth retardation (*p* = 0.001, OR: 0.16, 95% CI: 0.09–0.30), oral lesions (*p* = 0.005, OR: 0.39, 95% CI: 0.20–0.77), recurrent infections (*p* = 0.024, OR: 0.60, 95% CI: 0.38–0.94), and fever (*p* = 0.001, OR: 0.31, 95% CI: 0.15–0.63) were less common. The number of EIMs reported by the patients did not significantly differ between the groups (*p* > 0.05). CEAS/CNSU caused by *SLCO2A1* defects, as well as congenital/familial diarrhea associated with *SLC26A1*, *SLC9A3*, and *GUCY2C* mutations, were the most prevalent monogenic disorders in patients with LO-mIBD, significantly more common than in those with IO-mIBD (*p* < 0.001, Table 4). In contrast, IL-10 signaling deficiency was notably prevalent in patients with IO-mIBD, with no case reports in those with LO-mIBD (*p* < 0.001).

Multifactorial logistic regression analysis was conducted to evaluate the influence of confounding factors (Supplementary Table 4). The results remained statistically significant after adjusting for age and parental consanguinity. Adjustment for genotype (analysis based on cases within the 24 overlapping genes) altered the significance of features such as zygosity, GI complication of stricture, lesions in the duodenum and rectum, erosions, pseudopolyps, villous atrophy, and oral EIMs. This suggests that genotype plays a critical role in driving differences in these phenotypic features.

The percentage of missing data between the groups did not significantly differ in the above analysis (2.9–51.8% and 0–50% in the IO-mIBD and LO-mIBD groups, respectively, *p* > 0.05). The results of sensitivity analysis (Supplementary Table 6) revealed that the differences remained significant when the analysis was conducted using the assumed RI_LTFU/FU_, except for ulcers and pathological features, for which approximately 50% of the data were missing. Approximately 60–80% of the included cases were derived from high-quality case reports and case series (Supplementary Fig. 3a). WES (43.59%) was the sequencing technology most frequently used for diagnosis, followed by Sanger sequencing (28.2%) and TGPS/TNGS (16.3%, Supplementary Fig. 3c). The reevaluation of variant pathogenicity according to the latest ACMG guidelines showed that over 90% of the included cases had pathogenic and likely pathogenic variants (76.19% and 16.85%, respectively, Supplementary Fig. 3d). No significant differences were found in the genetic testing technologies or variant pathogenicity between the groups. These findings collectively suggest a low risk of bias from missing data and limited confounding effects resulting from the heterogeneity of the reports and genetic testing technologies.

**Table 2:** Comparison of gastrointestinal manifestations in patients with late-onset vs. infantile-onset mIBD

	LO-mIBD	IO-mIBD	OR (95% CI)	*P* value
	% (*n*/*N*^†^)	% (*n*/*N*^†^)		
**GI phenotypes**				< 0.001
CD	43.6 (48/110) ^**^	24.1 (105/436)	8.53 (4.02, 18.11)	< 0.001
UC	5.5 (6/110)	4.1 (18/436)	6.22 (1.99, 19.49)	0.002
IBDU/IC	0.9 (1/110)	3.9 (17/436)	1.10 (0.13, 9.20)	0.931
IBD^‡^	8.2 (9/110) ^**^	38.5 (168/436)	(Ref)	(Ref)
Others^§^	41.8 (46/110) ^**^	29.4 (128/436)	6.71 (3.17, 14.21)	< 0.001
**GI symptoms**				
Abdominal pain	46.2 (43/93)	15.7 (54/344)	4.62 (2.80, 7.62)	< 0.001
Diarrhea	66.7 (62/93)	92.4 (318/344)	0.16 (0.09, 0.29)	< 0.001
Hematochezia	31.2 (29/93)	60.8 (209/344)	0.29 (0.18, 0.48)	< 0.001
Perianal disease	10.6 (10/94)	53.7 (202/376)	0.10 (0.05, 0.20)	< 0.001
**GI complications**				
Intestinal fistula	2.2 (2/89)	5.7 (19/335)	0.38 (0.09, 1.67)	0.294
Perforation	4.5 (4/89)	7.5 (25/335)	0.58 (0.20, 1.72)	0.324
Stricture	31.5 (28/89)	17.0 (57/335)	2.24 (1.32, 3.81)	0.002
**Lesion sites**				
Esophagus/Stomach	23.6 (21/89)	19.0 (65/343)	1.32 (0.76, 2.31)	0.328
Duodenum	20.2 (18/89)	25.1 (86/343)	0.76 (0.43, 1.34)	0.340
Small intestine	58.4 (52/89)	29.4 (101/343)	3.37 (2.08, 5.45)	< 0.001
Colon	62.9 (56/89)	87.2 (299/343)	0.25 (0.15, 0.43)	< 0.001
Rectum	11.2 (10/89)	22.2 (76/343)	0.45 (0.22, 0.90)	0.021
Upper GI involvement^¶^	31.5 (28/89)	31.2 (107/343)	1.01 (0.61, 1.67)	0.962
**Endoscopic findings**				
Ulcer	90.9 (60/66)	75.7 (159/210)	3.21 (1.31, 7.86)	0.008
Erosion	18.2 (12/66)	22.9 (48/210)	0.75 (0.37, 1.52)	0.422
Pseudopolyps	4.5 (3/66)	18.6 (39/210)	0.21 (0.06,0.70)	0.006
**Pathological features**				
Villous atrophy	5.5 (3/55)	22.9 (52/236)	0.20 (0.06, 0.68)	0.005
Chronic active enteritis	63.6 (35/55)	71.2 (168/236)	0.71 (0.38, 1.31)	0.272
Apoptosis or epithelial injury	7.3 (4/55)	13.6 (32/236)	0.50 (0.17, 1.48)	0.202
Eosinophil-rich	7.3 (4/55)	8.1 (19/236)	0.90 (0.29, 2.75)	1.000
Lymphocytic	20.0 (11/55)	9.3 (22/236)	2.43 (1.10, 5.38)	0.025
Granulomatous	21.8 (12/55)	10.6 (25/236)	2.36 (1.10, 5.05)	0.024

CD, Crohn’s disease; UC, ulcerative colitis; IBDU, IBD-unclassified; IC, indeterminate colitis; GI, gastrointestinal tract

^†^. n = number of cases presenting the corresponding features; N = number of cases with available information about the features in the reports

^‡^. Gastrointestinal phenotypes labeled as IBD without specifying the exact subtype

^§^. Other chronic intestinal inflammation resembling IBD, see detailed definitions in Supplementary Table 1B

^¶^. The upper gastrointestinal tract includes the esophagus, stomach, and duodenum

^**^. Bonferroni-adjusted *P* < 0.05 between LO-mIBD and IO-mIBD

**Table 3:** Comparison of extraintestinal manifestations and comorbidities in patients with late-onset vs. infantile-onset mIBD

	LO-mIBD	IO-mIBD	OR (95% CI)	*P* value
	% (*n*/*N*^†^)	% (*n*/*N*^†^)		
**Extraintestinal manifestations**				
>1 EIMs	49.1 (54/110)	51.3 (217/423)	0.92 (0.60, 1.39)	0.680
>2 EIMs	29.1 (32/110)	26.5 (112/423)	1.14 (0.72, 1.81)	0.582
Anemia	30.9 (34/110)	18.4 (78/423)	1.98 (1.23, 3.18)	0.004
Growth retardation	11.8 (13/110)	44.9 (190/423)	0.16 (0.09, 0.30)	< 0.001
Oral lesions	10.0 (11/110)	22.0 (93/423)	0.39 (0.20, 0.77)	0.005
Skin lesions	29.1 (32/110)	36.9 (156/423)	0.70 (0.45, 1.11)	0.128
Musculoskeletal abnormality	29.1 (32/110)	10.6 (45/423)	3.45 (2.06, 5.77)	< 0.001
Hepato-splenic-biliary abnormality	13.6 (15/110)	13.9 (59/423)	0.97 (0.53, 1.79)	0.933
Chronic renal disease	1.8 (2/110)	1.9 (8/423)	0.96 (0.20, 4.59)	1.000
Chronic pulmonary disease	7.3 (8110)	3.3 (14/423)	2.29 (0.94, 5.61)	0.111
Cardiovascular abnormality	3.6 (4/110)	4.3 (18/423)	0.85 (0.28, 2.56)	0.983
Endocrine abnormality	5.5 (6/110)	4.0 (17/423)	1.38 (0.53, 3.58)	0.692
Hematological abnormality	10.9 (12/110)	7.6 (32/423)	1.50 (0.74, 3.01)	0.256
Neurological abnormality	9.1 (10/110)	5.4 (23/423)	1.74 (0.80, 3.77)	0.157
Ocular and auditory abnormality	1.8 (2/110)	2.8 (12/423)	0.63 (0.14, 2.88)	0.794
Lymphadenopathy	7.3 (8/110)	4.0 (17/423)	1.87 (0.79, 4.46)	0.150
**Comorbidities**				
Recurrent infections	30.0 (33/110)	41.8 (177/423)	0.60 (0.38, 0.94)	0.024
Relapsing fever	8.2 (9/110)	22.5 (95/423)	0.31 (0.15, 0.63)	0.001
Autoimmune disease	10.9 (12/110)	9.0 (38/423)	1.24 (0.63, 2.46)	0.537
Autoinflammation	8.2 (9/110)	10.9 (46/423)	0.73 (0.35, 1.54)	0.408
HLH/MAS	0.9 (1/110)	3.3 (14/423)	0.27 (0.04, 2.06)	0.302
Allergy	0.9 (1/110)	2.4 (10/423)	0.38 (0.05, 2.99)	0.562
Ectodermal dysplasia	3.6 (4/110)	5.7 (24/423)	0.63 (0.21, 1.85)	0.394
Dysmorphism	3.6 (4/110)	6.4 (27/423)	0.55 (0.19, 1.62)	0.273
Malignancy	5.5 (6/110)	3.8 (16/423)	1.47 (0.56, 3.84)	0.606

^†^. n = number of cases presenting the corresponding features; N = number of cases with available information about the features in the reports

EIMs, extraintestinal manifestations; HLH, hemophagocytic lymphohistiocytosis; MAS, macrophage activation syndrome

**Table 4:** Prevalence of monogenic disorders in patients with late-onset and infantile-onset mIBD

Monogenic disorders	Pathogenic genes	LO-mIBD *n* (%) *N* = 110	IO-mIBD *n* (%) *N* = 436	*P* value
IL-10 signaling deficiency	*IL10*,* IL10RA*,* IL10RB*	0 (0.0)	156 (35.8)	< 0.001
IPEX or IPEX-like	*IL2RA*,* MALT1*,* FOXP3*,* BACH2*,* LRBA*,* CTLA4*,* STAT1*,* STAT3*,* CARD11*,* ITCH*	11 (10.0)	55 (12.6)	0.516
CGD	*CYBB*,* CYBA*,* CYBC1*,* NCF1*,* NCF2*,* NCF4*	11 (10.0)	27 (6.2)	0.206
CEAS/CNSU	*SLCO2A1*	26 (23.6)	5 (1.2)	< 0.001
XLP2	*XIAP*	5 (4.6)	15 (3.4)	0.789
SD/THES	*SKIV2L*,* TTC37*	0 (0.0)	16 (3.67)	0.085
Congenital/familial diarrhea	*SLC26A3*,* SLC9A3*,* GUCY2C*	12 (10.9)	4 (0.9)	< 0.001
HA20	*TNFAIP3*	4 (3.6)	11 (2.5)	0.755
WAS or WAS-like	*WAS*,* ARPC1B*	1 (0.9)	10 (2.3)	0.587

Only monogenic disorders or syndromes with a total of more than 10 included cases were listed. Abbreviations: IL-10: interleukin-10; IPEX: X-linked immune dysregulation, polyendocrinopathy, enteropathy syndrome; CGD, chronic granulomatous disease; CEAS, chronic enteropathy associated with the *SLCO2A1* gene; CNSU, chronic nonspecific multiple ulcers of the small intestine; XLP2, X-linked lymphoproliferative syndrome 2; SD/THES, syndromic diarrhea/tricho-hepato-enteric syndrome; HA20, haploinsufficiency of A20; WAS, Wiskott–Aldrich syndrome;

### Genotype-associated clinical manifestations and treatment of late-onset and infantile-onset mIBD

Given the heterogeneity and phenotype-genotype associations of mIBD, it is necessary to investigate the clinical manifestations from the perspective of genotypes to further understand the phenotypic characteristics of LO-mIBD. Due to the complex genotypes and dispersed cases (Supplementary Fig. 4), we focused on the top 14 common pathogenic genes in each group, which accounted for the majority of cases (72.21% and 74.55% in the IO-mIBD and LO-mIBD groups, respectively). Three genes—*CYBB*, *XIAP*, and *TNFAIP3*—overlapped between the groups.

**Fig. 2:**
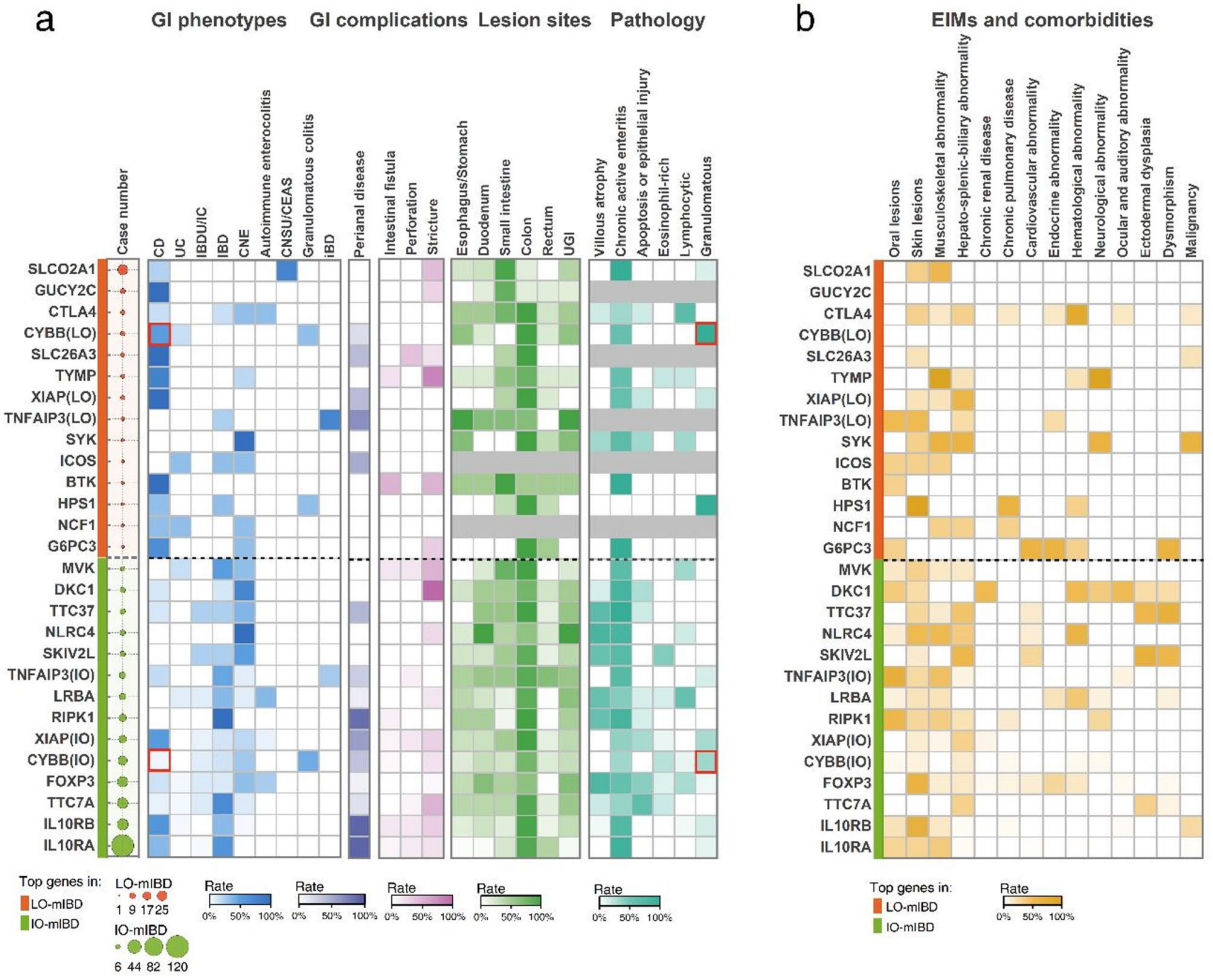
Clinical manifestations in patients with late-onset and infantile-onset mIBD stratified by the 14 most common gene defects. (**a**) The number of cases and GI manifestations stratified by gene defects. Red and green bars denote the 14 most prevalent pathogenic genes in patients with LO-mIBD and IO-mIBD, respectively. Dot size indicates the number of cases within each gene, with red and green dots representing the LO-mIBD and IO-mIBD cases, respectively. The color intensity of the square represents the incidence of the corresponding features, from 0% to 100%, whereas gray indicates unavailable data. The red border indicates a significant difference between patients with IO-mIBD and LO-mIBD caused by the same gene defects (*p* < 0.05). (**b**) Extraintestinal manifestations and comorbidities stratified by gene defects. The color intensity of the square represents the incidence of the corresponding features, ranging from 0% to 100%. Abbreviations: CD, Crohn’s disease; UC, ulcerative colitis; IBDU, IBD-unclassified; IC, indeterminate colitis; IBD, refers to the category of IBD without delineating specific subtypes in this study; UGI: upper gastrointestinal tract, mainly in anatomical terms; CNE, chronic nonspecific enterocolitis; CNSU, chronic nonspecific multiple ulcers of the small intestine; CEAS, chronic enteropathy associated with the *SLCO2A1* gene; iBD, intestinal Behçet’s disease; EIMs, extraintestinal manifestations

Figure 2 shows the clinical manifestations stratified by the 14 common genes in each group. Consistent with the above observations, the GI lesions in LO-mIBD with various monogenetic deficiencies were predominantly characterized by a CD-like phenotype, particularly in cases caused by *GUCY2C*, *SLC26A3*, *XIAP*, *BTK*, *TYMP*, *G6PC3*, and *CYBB* mutations, with a proportion of no less than 50%. In contrast, IBD and chronic nonspecific enterocolitis (CNE) were the main phenotypes associated with several common monogenic defects in those with IO-mIBD. In addition to the CD-like phenotype, patients with LO-mIBD caused by *ICOS* deficiency could be diagnosed with UC, IBD, and CNE; those with TNFAIP3 deficiency could be diagnosed with IBD and intestinal Behçet’s disease (iBD); and those with SYK mutations were characterized by CNE. Intestinal strictures were notably prevalent in patients with LO-mIBD, particularly in those caused by *TYMP*, *BTK*, *SLCO2A1*, *G6PC3*, *GUCY2C*, and *SLC26A3* mutations. The EIMs and comorbidities in IO-mIBD and LO-mIBD were found to be complex and multi-systemic, depending on the specific genes involved (Fig. 2b).

Regarding the three genes that were common in both groups, *CYBB* deficiency usually leads to granulomatous colitis with perianal disease, which is often diagnosed as CD or chronic granulomatous disease (CGD). However, the initial diagnosis of CD (50.0% vs. 4.8%, *p* < 0.05) and pathological findings of granulomas (100.0% vs. 33.3%, *p* = 0.029) were more prominent in patients with LO-mIBD than in those with IO-mIBD. *TNFAIP3* deficiency typically causes intestinal, oral, and genital ulcers, which are frequently diagnosed as iBD or CD. Patients with LO-mIBD were more likely to be diagnosed with iBD than those with IO-mIBD (75.0% vs. 27.3%), although this difference was not statistically significant (*p* > 0.05). *XIAP* deficiency is typically characterized by CD-like enteropathy and perianal disease.

**Fig. 3:**
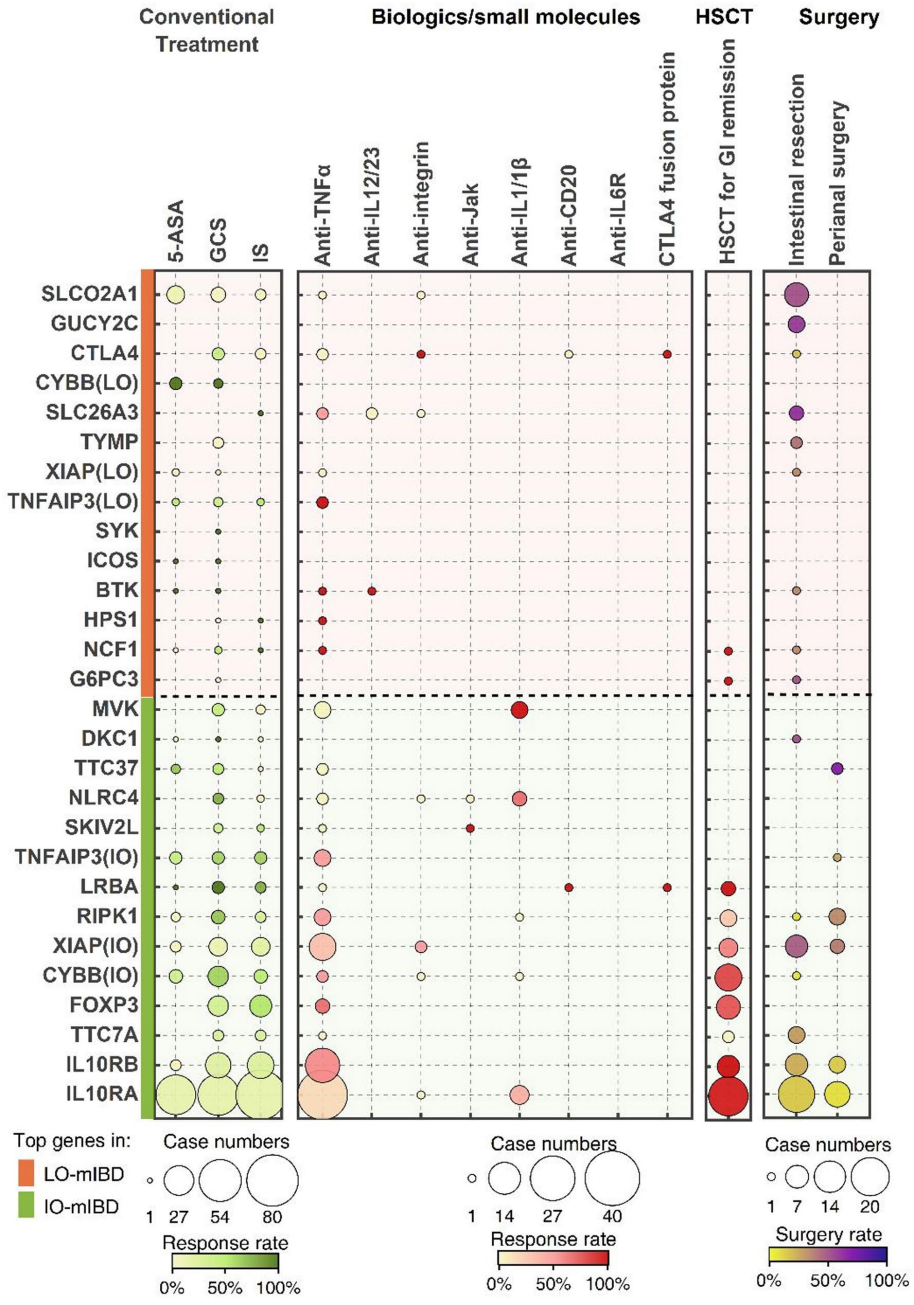
Treatment interventions and responses in late-onset and infantile-onset mIBD stratified by gene defects. Stratification of the therapeutic response to conventional treatment, biologics/small molecules, and HSCT, as well as the surgical rates in patients with IO-mIBD and LO-mIBD according to the 14 most common gene defects. Red and green bars denote the 14 most prevalent pathogenic genes in patients with LO-mIBD and IO-mIBD, respectively. The dot size represents the number of patients receiving treatment. The color intensity indicates the response rate to treatment, ranging from 0% to 100%. Abbreviations: 5-ASA, 5-aminosalicylic acid; GCS, glucocorticoids; IS, immune suppressants and modulators, including azathioprine, methotrexate, cyclosporine, thalidomide, tacrolimus, and sirolimus; HSCT, hematopoietic stem cell transplantation

The treatment choices differed somewhat between patients with IO-mIBD and LO-mIBD (Supplementary Table 7). Surgical resection was significantly more prevalent in patients with LO-mIBD, particularly in those caused by *SLC26A3*, *GUCY2C*, *SLCO2A1*, and *G6PC3* mutations, with a resection rate of approximately 50.0%. The resection rates remained statistically significant after adjusting for GI complications or genotypes in the multifactorial regression analysis (Supplementary Table 5). HSCT was performed in 26.5% of IO-mIBD cases, providing intestinal benefits for those with certain monogenic defects, such as *IL10RA/B*, *CYBB*, *XIAP*, and *LRBA* deficiencies. However, only three cases of LO-mIBD—with *G6PC3*, *NCF1*, and *LRBA* mutations, respectively—underwent HSCT and showed GI improvement. These results are exploratory due to the small sample size and need to be confirmed by future studies. The response to traditional medications in patients with mIBD varied primarily according to the underlying monogenic defects (Fig. 3). However, among patients with *CYBB* deficiency, those with LO-mIBD had higher response rates to 5-aminosalicylic acid (5-ASA, 100.0% vs. 33.3%) and glucocorticoids (GCS, 100.0% vs. 66.7%) than those with IO-mIBD, although the difference did not reach significance (*p* > 0.05). Some other treatments displayed GI effectiveness in certain monogenic defects with limited case numbers, such as anti-TNFα biologics for patients with LO-mIBD caused by BTK, NCF1, and TNFAIP3 deficiencies; anti-IL12/23 biologics for those caused by BTK deficiency; CTLA4 fusion protein (abatacept) for those caused by *LRBA* and *CTLA4* deficiencies; and anti-IL-1β/1R biologics for patients caused by mutations of *IL10RA*, *NLRC4*, and *MVK*, etc.

### Gene function and intestinal pathogenesis underlying monogenetic defects in late-onset mIBD compared with infantile-onset mIBD

The age of onset of IBD-like phenotypes varies with the gene, suggesting an association between the pathogenesis of gene defects and the age of onset. It remains unclear whether the pathogenic mechanisms of genetic defects between IO-mIBD and LO-mIBD are similar or distinct. The GI pathogenesis of mIBD primarily involves defects in immune function and epithelial maintenance. We thus compared the pathogenic mechanisms and immune-related manifestations based on the 14 most common gene defects in each group.

**Fig. 4:**
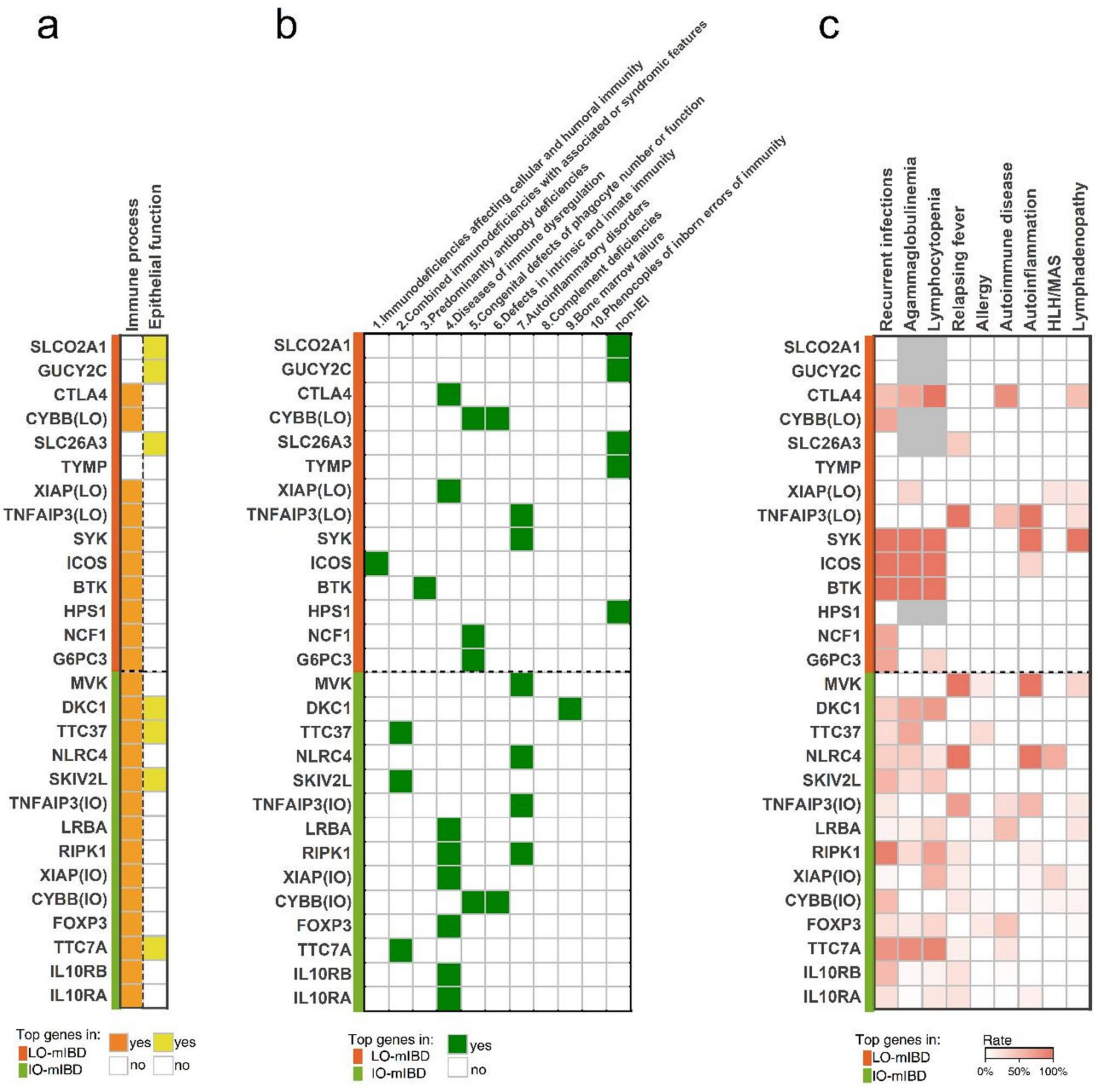
Pathogenesis and immune-associated abnormalities of common gene defects in patients with late-onset and infantile-onset mIBD. (**a**) The involvement of the immune process and epithelial function of the top 14 common genes in patients with IO-mIBD and LO-mIBD, respectively. The blank boxes indicate no, whereas the orange and yellow boxes indicate yes. (**b**) The categories of inborn errors of immunity (IEI) classification of gene defects, according to the 2022 classification from the International Union of Immunological Societies (IUIS). Non-IEI refers to genes that are not included in the IEI classification. The blank and green boxes indicate no and yes, respectively. (**c**) The incidence of immune-related abnormalities stratified by gene defects. The color intensity represents the incidence ranging from 0% to 100%, with gray indicating unavailable data. Abbreviations: HLH, hemophagocytic lymphohistiocytosis; MAS, macrophage activation syndrome

All of the 14 most common genes in the IO-mIBD group were functionally related to the immune process (Fig. 4a), and their deficiencies are included in the latest IEI classification, with “diseases of immune dysregulation” being the most common IEIs/PIDs category (Fig. 4b). Patients with IO-mIBD caused by these gene defects usually exhibited immune abnormalities such as recurrent infections, agammaglobulinemia, lymphocytopenia, autoimmune disease, and autoinflammation (Fig. 4c). In addition, genes such as *TTC7A*, *SKIV2L*, *TTC37*, and *DKC1* also play a role in epithelial maintenance, with their defects often leading to ectodermal dysplasia, dysmorphism, and intestinal epithelial damage (Fig. 2b).

For the top 14 gene defects in the LO-mIBD group, the most common IEIs/PIDs category was “congenital defects of phagocyte number or function”, involving *G6PC3*, *CYBB*, and *NCF1* deficiencies (Fig. 4b). In addition, several gene defects have not been included in the IEIs/PIDs classification, including *SLCO2A1*, *GUCY2C*, *SLC26A3*, *TYMP*, and *HPS1* mutations. The proportion of non-IEI gene defects was significantly higher in the LO-mIBD group than in the IO-mIBD group (*p* < 0.05). Patients with LO-mIBD caused by these gene mutations rarely present with immune abnormalities (Fig. 4c). Furthermore, these non-IEI gene defects can be pathogenic via nonimmune processes, particularly epithelial dysfunction. For example, *SLC26A3* and *GUCY2C* mutations impair epithelial ion transport, while *SLCO2A1* mutations disrupt prostaglandin transport and compromise the intestinal barrier (Supplementary Table 8). The results of GO enrichment analysis of the common genes specific to each group further supported these findings. GO terms associated with immune and inflammatory regulation were significantly enriched in the IO-mIBD group, whereas those related to the response to pathogens and ion transport were enriched in the LO-mIBD group (*p* < 0.05; Supplementary Table 9).

## Discussion

LO-mIBD remains underexplored despite its increasing relevance in clinical practice, particularly in adult gastroenterology. This study provides the first comprehensive evaluation of the LO-mIBD population with IBD-like phenotypes developing after 16 years of age, revealing some distinct clinical and genetic features compared with IO-mIBD cases. Our findings bridge the knowledge gaps regarding LO-mIBD and provide novel insights to facilitate clinical management and future research.

The genetic landscape of mIBD follows Mendelian inheritance patterns, as reflected in family history, consanguinity, zygosity types, and inheritance modes [1, 9]. Both patients with LO-mIBD and IO-mIBD had high rates of positive family history and were predominantly affected by homozygous and autosomal recessive mutations, consistent with the findings of Nambu et al. [9]. However, patients with LO-mIBD had less parental consanguinity and more heterozygous mutations in autosomal dominant genes (e.g., *GUCY2C*, *CTLA4*, *SYK*) than those with IO-mIBD. These findings highlight the importance of genetic counseling for patients with LO-mIBD, as autosomal dominant mutations confer a 50% risk of transmission to their offspring.

The GI phenotypes of patients with mIBD are highly heterogeneous [1]. A previous study of all published cases of mIBD has revealed that most cases were classified as IBD without specifying the subtype, followed by CD [9]. However, data specifically on late-onset populations are lacking. Our study revealed that the CD-like phenotype was the most common presentation in patients with LO-mIBD and was more prevalent than in those with IO-mIBD. These findings are consistent with those of Crowley et al. [23], who reported CD-like phenotypes in the majority of late-onset mIBD cases diagnosed during late childhood and early adolescence. Importantly, our findings confirm and extend the previous results of CD-like phenotypes in late-onset mIBD populations by showing higher rates of intestinal ulcers, strictures, small bowel lesions, and granulomas in LO-mIBD.

The clinical manifestations of patients with LO-mIBD are largely influenced by genotype, as demonstrated in this study. The stratified analysis revealed that certain genetic defects, such as *GUCY2C*, *SLC26A3*, *XIAP*, *BTK*, *TYMP*, *G6PC3*, and *CYBB* deficiencies, were commonly identified in patients with LO-mIBD and frequently result in CD-like phenotypes. Additionally, monogenic disorders such as CEAS and familial/congenital diarrhea were prominent in patients with LO-mIBD. CEAS, typically observed in adolescents and adults with *SLCO2A1* deficiency, mimics CD with severe and multiple small intestinal ulcers [29, 30]. Patients with congenital diarrhea (*SLC26A3* or *SLC9A3* mutations) and familial diarrhea (*GUCY2C* mutations) typically experience severe diarrhea and electrolyte imbalances early in life, with intestinal inflammation and obstruction often developing in late childhood or adulthood [31, 32]. These monogenic etiologies should be prioritized in patients with late-onset and refractory CD-like phenotypes, and adult gastroenterologists should fully understand the systemic manifestations and optimize clinical management.

Although most LO-mIBD cases exhibit features overlapping with CD, some distinct endoscopic, pathological, and imaging characteristics have been noted in certain patients with mIBD, which may aid in distinguishing them from those with classic IBD [20, 33, 34]. In addition, it is also important to recognize that patients with mIBD present with various GI phenotypes, even in late-onset populations. Examples include iBD-like isolated and round ulcers in those with haploinsufficiency of A20 (HA20) [35, 36] and celiac-like villous atrophy and lymphocyte infiltration in those with X-linked immune dysregulation, polyendocrinopathy, enteropathy syndrome (IPEX) or IPEX-like syndromes [37, 38]. Therefore, the GI phenotypes in late-onset mIBD should be comprehensively evaluated by integrating the details of endoscopic findings, histopathology, other laboratory tests, and systemic manifestations. Future research should be dedicated to clarifying the GI phenotypic spectrum to help optimize disease classification and enhance understanding of LO-mIBD. In addition, given the complex and genotype-driven systemic manifestations of mIBD, a multidisciplinary strategy is recommended for adult gastroenterologists in the screening and management of late-onset mIBD.

The clinical management of LO-mIBD remains complex, as patients with LO-mIBD also exhibit refractoriness to traditional treatment, which varies by genotype. In addition, those with *CYBB* defects present with milder phenotypes and better GI responses in LO-mIBD than in IO-mIBD, consistent with prior reports on *CYBB*-associated CGD colitis [38]. However, these differences may be influenced by factors such as drug dose, administration mode, and follow-up duration. Higher surgical resection rates in patients with LO-mIBD may reflect delayed diagnosis, more insidious disease progression, or reduced responsiveness to medications. For example, patients with *SLCO2A1* or *TYMP* defects respond poorly to conventional therapy [39, 40], while those with *GUCY2C* defects may experience cumulative intestinal damage over time [31]. Variations in follow-up durations and in treatment strategies between pediatric and adult patients may further impact the outcomes, as surgical rates may increase over follow-up time and surgery is generally less recommended in children than in adults [41]. Nonetheless, due to the limited sample size—particularly for those undergoing HSCT—these findings regarding treatment and effectiveness remain exploratory and warrant validation in larger prospective cohorts.

In mIBD, the intestinal disease typically arises from immune and epithelial dysfunctions that vary by underlying genetic defects and shape both the clinical phenotype and treatment response [6, 42, 43]. In this study, LO-mIBD was more frequently associated with phagocyte-related immunodeficiencies and non-immune epithelial defects, while IO-mIBD was mostly linked to immune dysregulation pathways. This is consistent with a previous study that highlighted immunodeficiencies in earlier-onset mIBD populations [44]. Correspondingly, patients with LO-mIBD had significantly fewer recurrent infections and fevers than those with IO-mIBD, further supporting distinct immune pathogenic mechanisms between the two groups. Age-related variation in immune development may also play a role. These findings indicate that mIBD should be considered even in late-onset cases with less pronounced signs of immunodeficiency or infection susceptibility, despite prior studies emphasizing recurrent infections in very early-onset mIBD populations [45, 46]. Notably, LO-mIBD patients with phagocyte- or epithelial-related defects respond poorly to conventional therapies and lack effective alternatives [42, 47]. Targeting these pathways may hold therapeutic potential. Further functional studies are needed to confirm this hypothesis and support the development of targeted treatments.

This study focuses on late-onset mIBD for the first time. However, as the understanding of mIBD evolves, the definition and research of late-onset mIBD require ongoing refinement. In this study, we comparatively analyzed the characteristics of LO-mIBD and IO-mIBD cases. However, the age of onset, as well as the clinical and genetic features of mIBD, may follow a continuum. Patients with an onset between 2 and 16 years may exhibit intermediate characteristics. Future studies including a wider age range could help better delineate the disease spectrum and age-related trends in mIBD. Furthermore, the advancement and increased accessibility of genetic testing could largely facilitate the identification of mIBD, especially for cases with milder manifestations and later onset [48]. The interpretation of genetic variants in different studies may have been influenced by the rapid advancements in human genetics over time. Consequently, the definition of late-onset mIBD cases may evolve continuously, requiring ongoing data updates, enhanced comparability across studies, and further exploration of the correlation among specific variants, pathogenicity, and clinical outcomes.

This study had some limitations. First, the retrospective nature of the published data inherently introduced biases of missing data, but our preliminary sensitivity analyses indicated minor risks. Second, reliance on published case reports and series may lead to selection or publication bias. These reports tend to highlight rare and severe cases; therefore, the prevalence of LO-mIBD may be underestimated. Third, the heterogeneity among reports—disease understanding, diagnostic criteria and tools, and healthcare resources differed across eras and regions—potentially skews results toward regions or periods with advanced genetic testing and specialized expertise and introduces a risk of misclassification of GI phenotypes. Fourth, reliance on binary or categorical outcomes may have constrained the ability to capture complex clinical processes and details. Due to heterogeneous data and a small sample size, the robustness of the stratified analysis was limited, and a multivariate model including all confounding factors was not possible yet, which warrants further investigation in future studies. Finally, due to the retrospective design, the direct applicability of these identified features in LO-mIBD screening is limited and requires validation in future cohort studies. Collectively, these limitations could be addressed by future large-scale, prospective, and standardized studies to validate and extend our findings.

In conclusion, mIBD is a clinical challenge across various age groups, including adult gastroenterology. Late-onset mIBD remains highly heterogeneous and difficult to manage. Although genomic sequencing advances have facilitated the identification and reporting of LO-mIBD cases, a deeper understanding of this disease subgroup is still needed to establish timely and effective clinical management strategies. Our study delineates the clinical spectrum and underlying pathogenic mechanisms of LO‑mIBD—particularly those associated with relatively common genetic defects—providing insights for clinical practice and further research. Future studies on LO-mIBD involving larger sample sizes, diverse age subgroups, prospective controlled cohorts, as well as functional investigations are essential to validate and extend these observations and to optimize patient management.

## Electronic supplementary material

Below is the link to the electronic supplementary material.

Supplementary Material 1

## Data Availability

The data analyzed for this study can be available from the corresponding author upon reasonable request.

## Abbreviations

CD: Crohn’s disease
CEAS: Chronic enteropathy associated with the SLCO2A1 gene
CGD: Chronic granulomatous disease
CI: Confidence interval
CNE: Chronic nonspecific enterocolitis
CNSU: Chronic nonspecific multiple ulcers of the small intestine
CMUSE: Cryptogenic multifocal ulcerous stenosing enteritis
EIMs: Extraintestinal manifestations
GCS: Glucocorticoids
GI: Gastrointestinal
GO: Gene ontology
HA20: Haploinsufficiency of A20
HSCT: Hematopoietic stem cell transplantation
HLH: Hemophagocytic lymphohistiocytosis
IBD: Inflammatory bowel disease
IBDU: Inflammatory bowel disease-unclassified
iBD: Intestinal Behçet’s disease
IC: Indeterminate colitis
IEIs: Inborn errors of immunity
IL-10: Interleukin-10
IO-mIBD: Infantile-onset monogenic inflammatory bowel disease
IQR: Interquartile range
IPEX: X-linked immune dysregulation, polyendocrinopathy, enteropathy syndrome
IS: Immune suppressant and modulators
LO-mIBD: Late-onset monogenic inflammatory bowel disease
MAS: Macrophage activation syndrome
mIBD: Monogenic inflammatory bowel disease
OR: Odds ratio
PIDs: Primary immunodeficiency disorders
SD/THES: Syndromic diarrhea/tricho-hepato-enteric syndrome
TGPS: Targeted gene panel sequencing
TNGS: Targeted next-generation sequencing
UC: Ulcerative colitis
UGI: Upper gastrointestinal tract
WAS: Wiskott-Aldrich syndrome
WES: Whole exome sequencing
WGS: Whole-genome sequencing
XLP2: X-linked lymphoproliferative syndrome 2
5-ASA: 5-aminosalicylic acid
