# Supplementary material for: Comprehensive evaluation of clinical phenotypes and pathogenic features in late-onset monogenic inflammatory bowel disease: a comparative study with infantile-onset cases
Source: BMC Gastroenterol. 2025 Jun 5;25:432. doi: 10.1186/s12876-025-04041-4 (PMC12139175; doi:10.1186/s12876-025-04041-4)
Supplement: Supplementary file 1 — Supplementary Material 1 [file 12876_2025_4041_MOESM1_ESM.pdf]

Supporting information

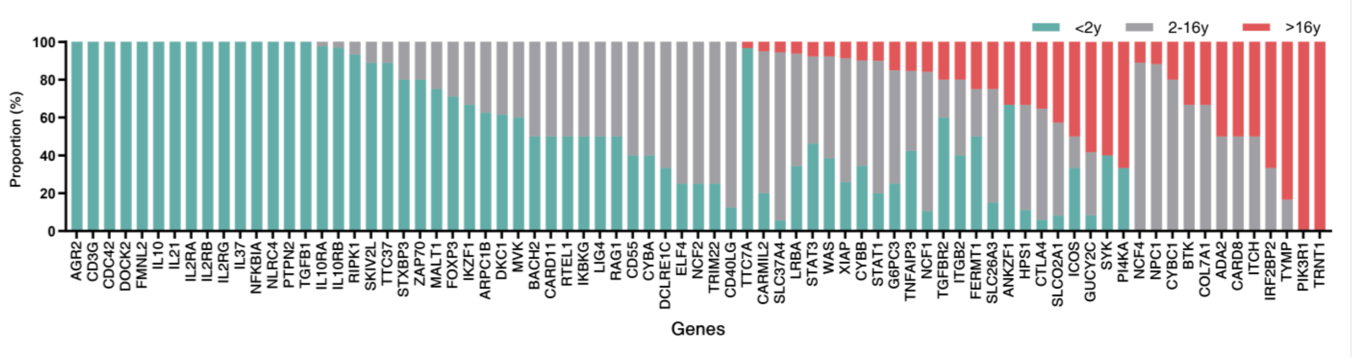

Supplementary Figure 1. Pathogenic genes and case distribution in infantile-onset and late-onset mIBD

A total of 77 pathogenic genes were identified in infantile-onset and late-onset mIBD cases, and the case proportion associated with each gene was analyzed. Green bars represent the proportion of patients with the onset of IBD-like phenotypes before age 2, while red bars indicate those with onset after age 16, respectively. Gray bars denote the remaining patients with onset between ages 2 and 16.

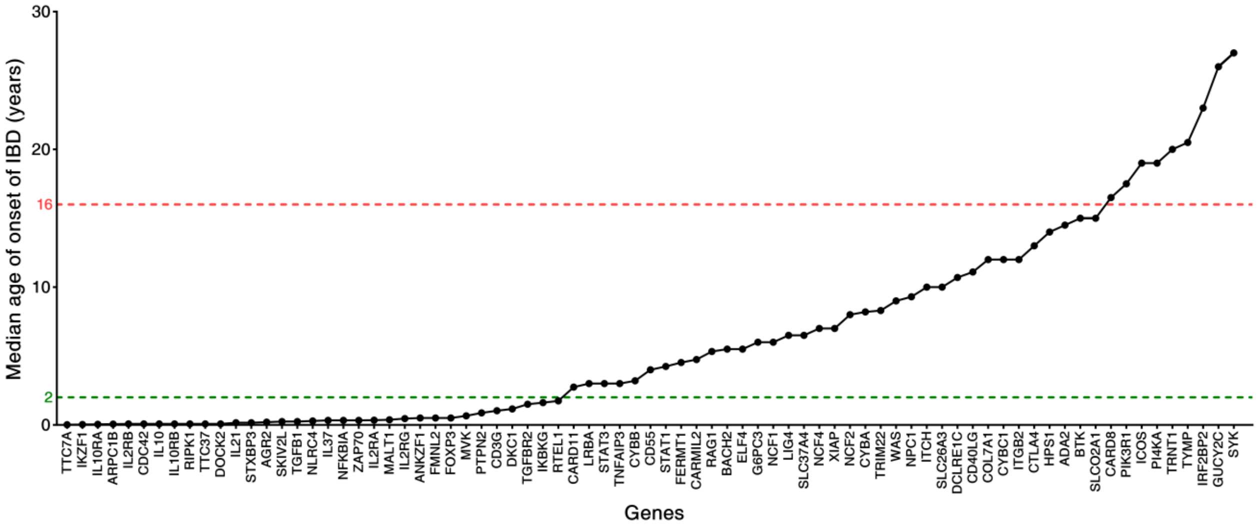

Supplementary Figure 2. Median age at onset of IBD-like phenotypes for each gene

The median age of onset of IBD-like manifestations for each gene was denoted by solid dots, calculated from all cases of mIBD screened in this (88 genes in total). The green and red lines represent the age thresholds of 2 years and 16 years, respectively.

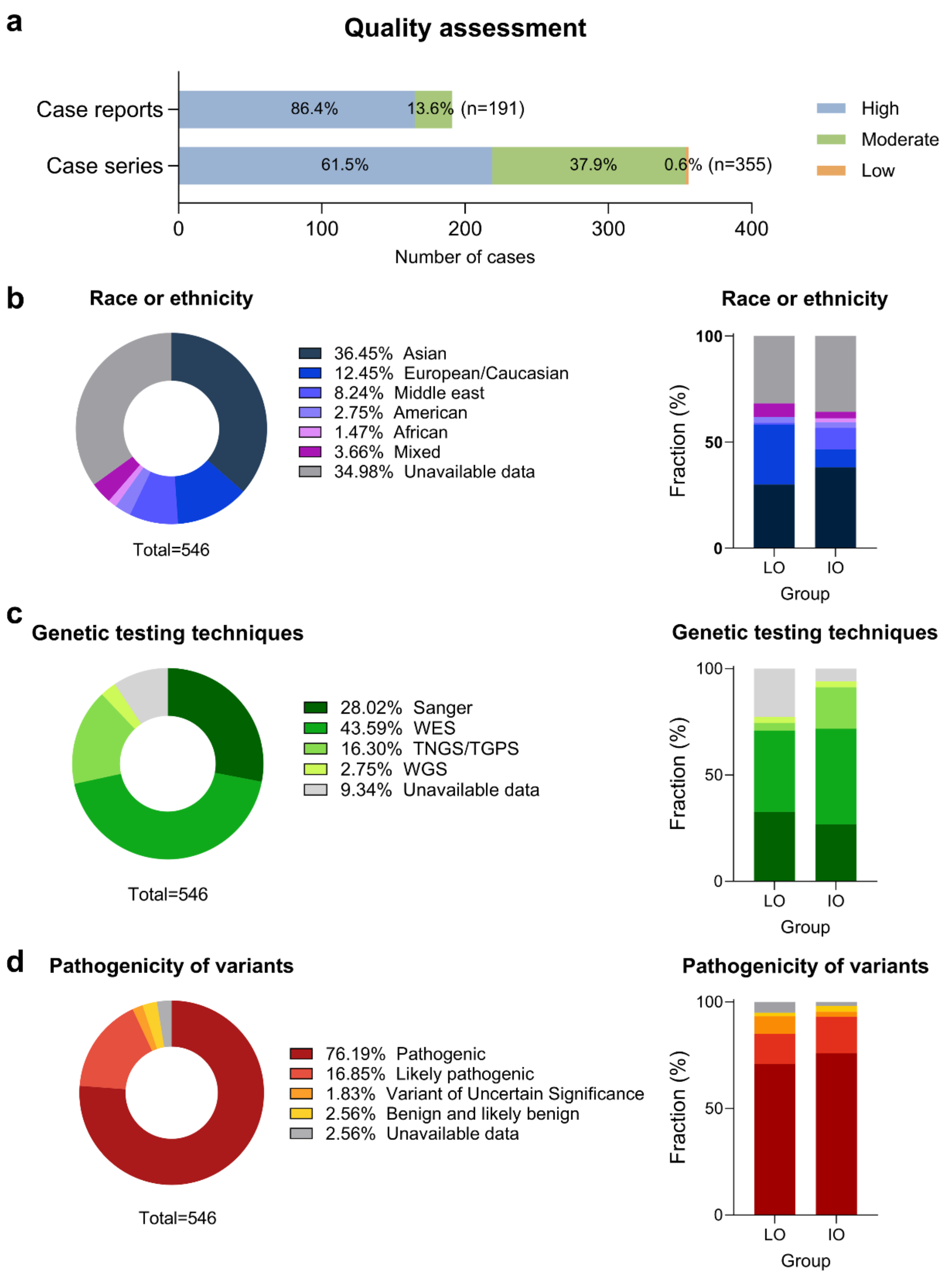

Supplementary figure 3. Additional information on the included cases.

(a) Quality assessment of 546 cases included in this study, based on the Joanna Briggs Institute (JBI) critical appraisal checklist for case reports and case series, respectively. Studies were categorized as high (≥7), moderate (4–6), and low quality (0–3). (b) Race or ethnicity distribution of the cases included in this study. (c) Genetic testing technologies employed in the cases included in this study. (d) Variant pathogenicity assessment of the cases, evaluated according to the American College of Medical Genetics and Genomics (ACMG) criteria. Abbreviations: WES, whole exome sequencing; TNGS, targeted next-generation sequencing; TGPS: targeted gene panel sequencing; WGS, whole genome sequencing.

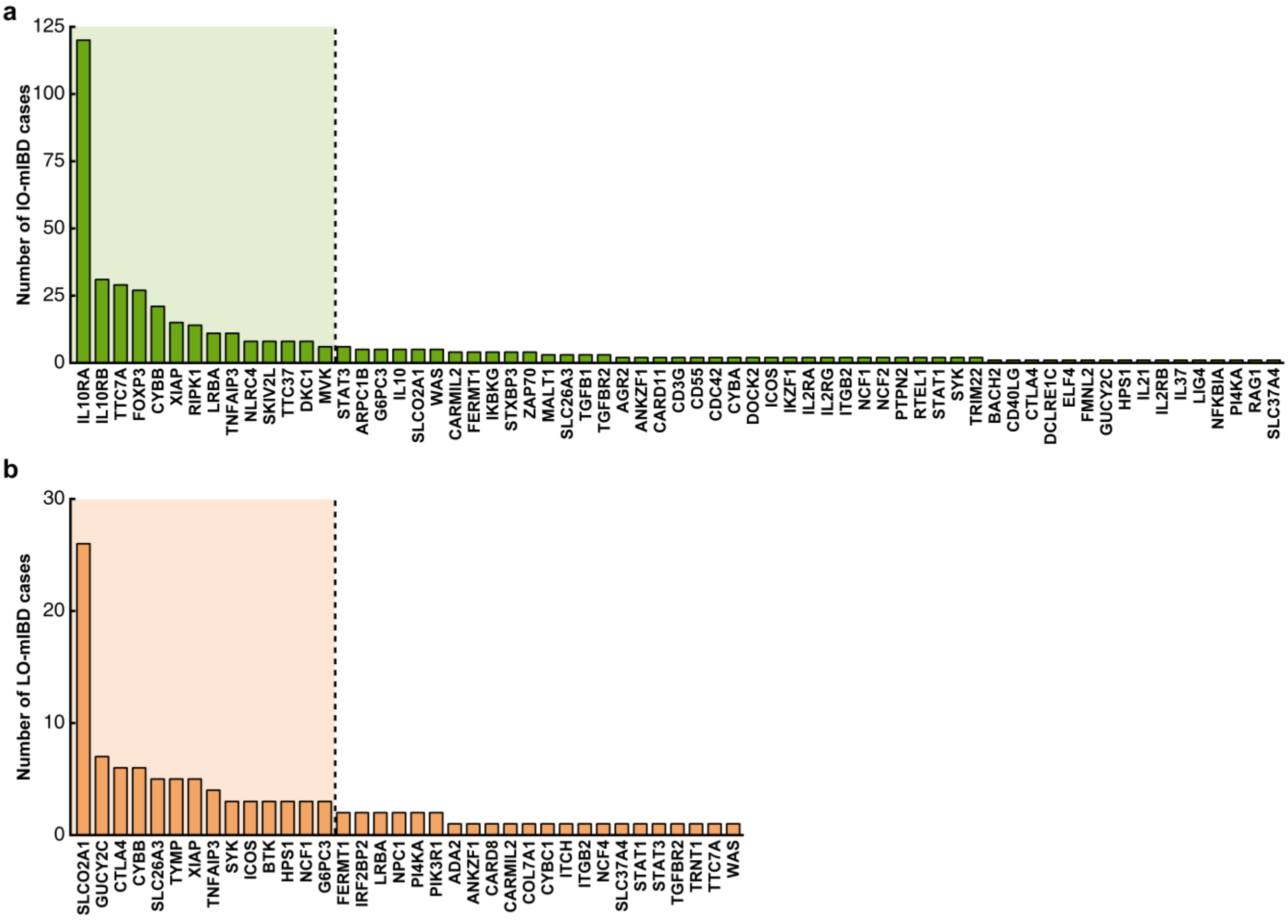

Supplementary Figure 4. Stratification and ranking of cases based on pathogenic genes in infantile-onset and late-onset mIBD

A total of 65 pathogenic genes were found in patients with infantile-onset mIBD (a) and 36 in late-onset mIBD (b). The case number for each gene defect is represented by the bars. The top 14 common genes in each group (highlighted) were chosen for further stratified analysis of features.

Supplementary Table1A. Categories and definitions of variant zygosity, referring to Klein, C. et al.[1]

Zygosity	Definition
Homozygotes	Both alleles of the gene are affected by the same variant
Compound heterozygotes (CH)	Two alleles of the gene are affected by different variants
Hemizygotes	One allele of the gene is affected while the other allele is absent or non-functional, observed in x-linked genes
Heterozygotes	Only one allele of the gene is affected

Supplementary Table1B. Categories and definitions of gastrointestinal phenotypes

GI phenotypes	Definition
CD	Crohn’s disease (-like)
UC	Ulcerative colitis (-like)
IBDU/IC	IBD-unclassified or indeterminate colitis (-like)
IBD	IBD (-like) without delineating specific subtypes
Others	Other chronic intestinal inflammation resembling IBD, including intestinal Behçet’s disease (iBD), chronic non-specific enterocolitis (CNE), autoimmune enteropathy, granulomatous colitis, chronic granulomatous disease (CGD) colitis, cryptogenic multifocal ulcerous stenosing enteritis (CMUSE), chronic enteropathy associated with the SLCO2A1 gene (CEAS), chronic non-specific multiple ulcers of the small intestine (CNSU), common variable immunodeficiency-associated enteropathy

Supplementary Table1C. Definition and criteria of gastrointestinal histopathological patterns, referring to Wilkins, B. J. et al.[2]

Intestinal pathologic patterns	Definition
Chronic active enteritis	Active inflammation (neutrophilic cryptitis and crypt abscesses) and chronic mucosal changes (crypt branching, crypt dropout, increased lymphoplasmacytic mucosal inflammation causing crypt liftoff)
Apoptosis or epithelial injury	Dilated with attenuated epithelial layers, loss of goblet or Paneth cells, crypt abscesses containing sloughed epithelial cells or apoptotic debris
Eosinophil-rich pattern	Lamina propria clustering and eosinophilic infiltration of surface and crypt epithelium
Lymphocytic pattern	Diffuse crypt and surface intraepithelial infiltration by lymphocytes, nodular lymphoid hyperplasia, lymphocyte or plasma cell depletion
Granulomatous pattern	Large and confluent sarcoid-like granulomas with multinucleated giant cells

Supplementary Table1D. Details for comorbidities assessed in this study

Category	Criteria or definition
Autoimmune disease	Autoimmune arthritis, thyroiditis, hepatitis, or pancreatitis; type 1 diabetes mellitus; psoriasis; systemic lupus erythematosus; Sjogren's syndrome; autoimmune hemolytic anemia, autoimmune neutropenia; immune thrombocytopenic purpura; glomerular nephropathy; nephrotic syndrome and autoimmune
Autoinflammation	Periodic fever of unknown origin; unexplained systemic inflammation of the skin, mucosa, serosa, or other organs; and persistent elevated inflammatory markers
Recurrent infections, fevers	Recurrent, severe, or opportunistic infections and fevers reported by authors based on clinical, laboratory, and other examing information
HLH/MAS	Hemophagocytic lymphohistiocytosis or macrophage activation syndrome reported by authors based on clinical, laboratory, and other examing information
Ectodermal dysplasia	Skin, hair, dental, or nail abnormalities
Dysmorphism	Dysmorphism in the face, limbs, or organs

Supplementary Table2A. JBI Critical Appraisal Checklist for Case Reports.

		Yes = 1	No/unclear = 0
1	Were patient's demographic characteristics clearly described?	□	□
2	Was the patient's history clearly described and presented as a timeline?	□	□
3	Was the current clinical condition of the patient on presentation clearly described?	□	□
4	Were diagnostic tests or assessment methods and the results clearly described?	□	□
5	Was the intervention(s) or treatment procedure(s) clearly described?	□	□
6	Was the post-intervention clinical condition clearly described?	□	□
7	Were adverse events (harms) or unanticipated events identified and described?	□	□
8	Does the case report provide takeaway lessons?	□	□
Score (0~3; low, 4~6:moderate; 7~8:high)			

Supplementary Table 2B. JBI Critical appraisal checklists for case series.

		Yes = 1	No/unclear = 0
1	Were there clear criteria for inclusion in the case series?	□	□
2	Was the condition measured in a standard, reliable way for all participants included in the case series?	□	□
3	Were valid methods used for identification of the condition for all participants included in the case series?	□	□
4	Did the case series have consecutive inclusion of participants?	□	□
5	Did the case series have complete inclusion of participants?	□	□
6	Was there clear reporting of the demographics of each participant in the study?	□	□
7	Was there clear reporting of clinical information of each participant?	□	□
8	Were the outcomes or follow up results of each case clearly reported?	□	□
9	Was there clear reporting of the presenting site(s)/clinic(s) demographic information?	□	□
10	Was statistical analysis appropriate?	□	□
Score (0~3; low, 4~6:moderate; 7~10:high)			

Supplementary Table 3. Univariate logistic regression of the variant zygosity in patients with late-onset and infantile onset mIBD

	OR (95% CI)	P value
**Homozygotes (Ref)**		
Heterozygotes	**2.564 (1.453, 4.523)**	**0.001**
Compound heterozygotes	0.683 (0.388, 1.203)	0.187
Hemizygotes	0.744 (0.392, 1.410)	0.365
**Compound heterozygotes (Ref)**		
Heterozygotes	**3.753 (1.948, 7.228)**	**<0.001**
Homozygotes	1.464 (0.831, 2.577)	0.187
Hemizygotes	1.089 (0.531, 2.234)	0.816
**Hemizygotes (Ref)**		
Heterozygotes	**3.446 (1.677, 7.083)**	**0.001**
Compound heterozygotes	0.918 (0.448, 1.884)	0.816
Homozygotes	1.344 (0.709, 2.549)	0.365
**Heterozygotes (Ref)**		
Compound heterozygotes	**0.266 (0.138, 0.513)**	**<0.001**
Hemizygotes	**0.290 (0.141, 0.596)**	**0.001**
Homozygotes	**0.390 (0.221, 0.688)**	**0.001**

Supplementary Table 4. Multivariate logistic regression analysis of demographic and clinical characteristics between LO-mIBD and IO-mIBD, adjusted for sex, consanguinity, and genotypes.

	Model 1		Model 2 (adjusted for sex)		Model 3 (adjusted for sex and consanguinity)		Model 4 (adjusted for genotypes)	
	OR (95% CI)	P value	OR (95%CI)	P value	OR (95% CI)	P value	OR (95% CI)	P value
**Sex (male)**	0.57 (0.38, 0.88)	**0.011**	-	-	-	-	0.60 (0.34, 1.03)	0.063
**Parental consanguinity**	0.49 (0.27, 0.88)	**0.015**	0.45 (0.25, 0.82)	**0.009**	-	-	0.57 (0.27, 1.21)	0.143
**Family history of similar monogenic disorders**	1.46 (0.92, 2.32)	0.108	1.45 (0.91, 2.31)	0.122	1.58 (0.96, 2.56)	0.063	1.03 (0.56, 1.86)	1.000
**Family history of IBD-like manifestations**	0.86 (0.52, 1.42)	0.553	0.84 (0.50, 1.40)	0.496	0.86 (0.51, 1.46)	0.570	0.75 (0.39, 1.42)	0.372
**Zygosity**								
Homozygotes (Ref)	-	-	-	-	-	-	-	-
Compound heterozygotes	0.68 (0.39, 1.20)	0.187	0.67 (0.37, 1.19)	0.167	0.519(0.249, 1.080)	0.079	0.91 (0.42, 1.95)	0.803
Heterozygotes	2.56 (1.45, 4.52)	**0.001**	2.51 (1.41, 4.45)	**0.002**	2.313(1.114, 4.802)	**0.024**	1.31 (0.65, 2.63)	0.45
Hemizygotes	0.74 (0.39, 1.41)	0.365	1.00 (0.50, 2.00)	0.989	0.982(0.426, 2.263)	0.966	0.41 (0.19, 0.89)	**0.024**
**Intestinal phenotypes**								
IBD‡ (Ref)	-	-	-	-	-	-	-	-
UC	6.22 (1.99, 19.49)	**0.002**	6.97 (2.16, 22.55)	**0.001**	10.37 (2.46, 43.79)	**0.001**	4.29 (1.16, 15.79)	**0.029**
CD	8.53 (4.02, 18.11)	**<0.001**	10.12 (4.58, 22.39)	**<0.001**	12.37 (4.99, 30.64)	**<0.001**	11.57 (4.41, 30.40)	**<0.001**
IBDU/IC	1.10 (0.13, 9.20)	0.931	1.78 (0.21, 15.49)	0.601	-	-	-	-
Others§	6.71 (3.17, 14.21)	**<0.001**	7.72 (3.50, 17.03)	**<0.001**	8.89 (3.62, 21.83)	**<0.001**	4.51 (1.85, 11.02)	**0.001**
**GI symptoms**								
Abdominal pain	4.62 (2.80, 7.62)	**<0.001**	4.50 (2.71, 7.45)	**<0.001**	6.35 (3.58, 11.27)	**<0.001**	3.04 (1.56, 5.93)	**0.001**
Diarrhea	0.16 (0.09, 0.29)	**<0.001**	0.17 (0.09, 0.31)	**<0.001**	0.12 (0.06, 0.24)	**<0.001**	0.45 (0.23, 0.89)	**0.021**
Hematochezia	0.29 (0.18, 0.48)	**<0.001**	0.30 (0.18, 0.49)	**<0.001**	0.25 (0.14, 0.45)	**<0.001**	0.40 (0.21, 0.74)	**0.003**

	Model 1		Model 2 (adjusted for sex)		Model 3 (adjusted for sex and consanguinity)		Model 4 (adjusted for genotypes)	
	OR (95% CI)	P value	OR (95%CI)	P value	OR (95% CI)	P value	OR (95% CI)	P value
Perianal disease	0.10 (0.05, 0.20)	**<0.001**	0.10 (0.05, 0.19)	**<0.001**	0.09 (0.04, 0.19)	**<0.001**	0.31 (0.14, 0.70)	**0.003**
**GI complications**								
Intestinal fistula	0.38 (0.09, 1.67)	0.294	0.37 (0.08, 1.62)	0.187	0.46 (0.10, 2.07)	0.313	-	0.506
Perforation	0.58 (0.20, 1.72)	0.324	0.58 (0.20, 1.72)	0.326	0.19 (0.03, 1.43)	0.106	0.78 (0.18, 3.38)	1.000
Stricture	2.24 (1.32, 3.81)	**0.002**	2.31 (1.35, 3.94)	**0.002**	2.13 (1.17, 3.88)	**0.013**	1.64 (0.78, 3.43)	0.191
**Lesion sites**								
Esophagus/Stomach	1.32 (0.76, 2.31)	0.328	1.34 (0.76, 2.38)	0.318	1.49 (0.79, 2.82)	0.221	0.86 (0.44, 1.69)	0.669
Duodenum	0.76 (0.43, 1.34)	0.340	0.79 (0.44, 1.40)	0.410	0.73 (0.39, 1.39)	0.340	0.49 (0.24, 0.98)	**0.041**
Small intestine	3.37 (2.08, 5.45)	**<0.001**	3.50 (2.15, 5.70)	**<0.001**	4.18 (2.41, 7.28)	**<0.001**	2.97 (1.61, 5.51)	**<0.001**
Colon	0.25 (0.15, 0.43)	**<0.001**	0.25 (0.15, 0.43)	**<0.001**	0.21 (0.12, 0.38)	**<0.001**	0.30 (0.15, 0.60)	**0.001**
Rectum	0.45 (0.22, 0.90)	**0.021**	0.42 (0.21, 0.86)	**0.018**	0.40 (0.19, 0.83)	**0.015**	0.49 (0.20, 1.16)	0.100
Upper GI involvement[¶]	1.01 (0.61, 1.67)	0.962	1.00 (0.60, 1.66)	0.983	1.02 (0.58, 1.80)	0.934	0.68 (0.37, 1.26)	0.221
**Endoscopic findings**								
Ulcer	3.21 (1.31, 7.86)	**0.008**	3.99 (1.50, 10.56)	**0.005**	5.16 (1.73, 15.34)	**0.003**	7.91 (2.58, 24.26)	**<0.001**
Erosion	0.75 (0.37, 1.52)	0.422	0.72 (0.35, 1.50)	0.381	0.69 (0.29, 1.46)	0.296	0.35 (0.14, 0.85)	**0.017**
Pseudopolyps	0.21 (0.06,0.70)	**0.006**	0.22 (0.07, 0.74)	**0.014**	0.15 (0.03, 0.63)	**0.010**	0.47 (0.12, 1.85)	0.269
**Pathological features**								
Villous atrophy	0.20 (0.06, 0.68)	**0.005**	0.20 (0.06, 0.68)	**0.009**	0.14 (0.03, 0.62)	**0.009**	0.39 (0.11, 1.44)	0.146
Chronic active enteritis	0.71 (0.38, 1.31)	0.272	0.74 (0.40, 1.39)	0.352	0.72 (0.35, 1.46)	0.362	1.46 (0.68, 3.12)	0.334
Apoptosis or epithelial injury	0.50 (0.17, 1.48)	0.202	0.50 (0.17, 1.49)	0.216	0.71 (0.23, 2.20)	0.550	0.49 (0.15, 1.59)	0.227
Eosinophil-rich	0.90 (0.29, 2.75)	1.000	0.97 (0.32, 3.00)	0.961	1.03 (0.32, 3.34)	0.963	0.43 (0.11, 1.59)	0.193
Lymphocytic	2.43 (1.10, 5.38)	**0.025**	2.39 (1.04, 5.46)	**0.039**	3.10 (1.21, 7.93)	**0.018**	1.84 (0.69, 4.85)	0.217
Granulomatous	2.36 (1.10, 5.05)	**0.024**	2.47 (1.14, 5.33)	**0.022**	2.63 (1.09, 6.35)	**0.032**	1.66 (0.64, 4.32)	0.297
**Extraintestinal manifestations**								

	Model 1		Model 2 (adjusted for sex)		Model 3 (adjusted for sex and consanguinity)		Model 4 (adjusted for genotypes)	
	**OR (95% CI)**	**P value**	**OR (95%CI)**	**P value**	**OR (95% CI)**	**P value**	**OR (95% CI)**	**P value**
> 1 EIMs	0.92 (0.60, 1.39)	0.680	0.85 (0.55, 1.30)	0.450	0.91 (0.57, 1.45)	0.692	1.02 (0.76, 1.37)	0.889
> 2 EIMs	1.14 (0.72, 1.81)	0.582	1.06 (0.66, 1.71)	0.813	1.15 (0.69, 1.91)	0.605	0.94 (0.59, 1.49)	0.790
Anemia	1.98 (1.23, 3.18)	**0.004**	1.84 (1.14, 2.98)	**0.013**	1.73 (1.02, 2.92)	**0.041**	1.55 (1.00, 2.39)	**0.049**
Growth retardation	0.16 (0.09, 0.30)	**<0.001**	0.15 (0.08, 0.29)	**<0.001**	0.14 (0.07, 0.28)	**<0.001**	0.17 (0.07, 0.42)	**<0.001**
Oral lesions	0.39 (0.20, 0.77)	**0.005**	0.39 (0.20, 0.76)	**0.006**	0.42 (0.21, 0.85)	**0.016**	0.57 (0.26, 1.23)	0.141
Skin lesions	0.70 (0.45, 1.11)	0.128	0.69 (0.43, 1.10)	0.115	0.64 (0.38, 1.07)	0.089	1.13 (0.73, 1.77)	0.586
Musculoskeletal abnormality	3.45 (2.06, 5.77)	**<0.001**	3.17 (1.88, 5.35)	**<0.001**	3.67 (2.06, 6.56)	**<0.001**	2.15 (1.20, 3.83)	**0.008**
Hepato-splenic-biliary abnormality	0.97 (0.53, 1.79)	0.933	1.01 (0.54, 1.86)	0.983	1.22 (0.63, 2.35)	0.577	0.68 (0.35, 1.33)	0.255
Chronic renal disease	0.96 (0.20, 4.59)	1.000	1.10 (0.23, 5.30)	0.908	1.02 (0.21, 4.98)	0.984	1.49 (0.09, 23.81)	1.000
Chronic pulmonary disease	2.29 (0.94, 5.61)	0.111	2.18 (0.88, 5.37)	0.091	2.73 (1.01, 7.42)	**0.049**	2.09 (0.69, 6.37)	0.306
Cardiovascular abnormality	0.85 (0.28, 2.56)	0.983	0.84 (0.28, 2.54)	0.751	0.77 (0.22, 2.75)	0.668	1.00 (0.29, 13.70)	1.000
Endocrine abnormality	1.38 (0.53, 3.58)	0.692	1.32 (0.47, 3.72)	0.595	1.18 (0.37, 3.78)	0.781	1.12 (0.40, 3.13)	0.827
Hematological abnormality	1.50 (0.74, 3.01)	0.256	1.57 (0.77, 3.18)	0.212	1.57 (0.72, 3.45)	0.259	1.35 (0.60, 3.07)	0.459
Neurological abnormality	1.74 (0.80, 3.77)	0.157	1.62 (0.74, 3.54)	0.228	1.85 (0.83, 4.12)	0.132	1.00 (0.29, 13.70)	1.000
Ocular and auditory abnormality	0.63 (0.14, 2.88)	0.794	0.66 (0.14, 3.00)	0.589	0.72, (0.15, 3.33)	0.669	0.37 (0.04, 3.29)	0.641
Lymphadenopathy	1.87 (0.79, 4.46)	0.150	2.03 (0.84, 4.91)	0.116	2.53 (0.99, 6.44)	0.052	1.33 (0.53, 3.31)	0.542
**Comorbidities**								
Recurrent infections	0.60 (0.38, 0.94)	**0.024**	0.59 (0.36, 0.93)	**0.024**	0.69 (0.42, 1.14)	0.143	0.57 (0.38, 0.85)	**0.004**
Relapsing fever	0.31 (0.15, 0.63)	**0.001**	0.30 (0.15, 0.63)	**0.001**	0.26 (0.11, 0.62)	**0.002**	0.42 (0.19, 0.93)	**0.023**
Autoimmune disease	1.24 (0.63, 2.46)	0.537	1.12 (0.57, 2.36)	0.668	1.21 (0.57, 2.58)	0.623	0.67 (0.32, 1.41)	0.286
Autoinflammation	0.73 (0.35, 1.54)	0.408	0.72 (0.34, 1.52)	0.385	0.80 (0.35, 1.82)	0.600	1.00 (0.42, 2.34)	0.993
HLH/MAS	0.27 (0.04, 2.06)	0.302	0.29 (0.04, 2.25)	0.237	0.36 (0.05, 2.89)	0.336	0.21 (0.03, 1.71)	0.210

	Model 1		Model 2 (adjusted for sex)		Model 3 (adjusted for sex and consanguinity)		Model 4 (adjusted for genotypes)	
	**OR (95% CI)**	**P value**	**OR (95%CI)**	**P value**	**OR (95% CI)**	**P value**	**OR (95% CI)**	**P value**
Allergy	0.38 (0.05, 2.99)	0.562	0.48 (0.06, 3.84)	0.488	0.58 (0.07, 4.89)	0.618	0.75 (0.07, 8.13)	1.000
Ectodermal dysplasia	0.63 (0.21, 1.85)	0.394	0.61 (0.21, 1.81)	0.375	0.53 (0.15, 1.82)	0.311	0.66 (0.21, 2.09)	0.480
Dysmorphism	0.55 (0.19, 1.62)	0.273	0.55 (0.19, 1.62)	0.277	0.67 (0.22, 2.02)	0.478	0.37 (0.08, 1.72)	0.319
Malignancy	1.47 (0.56, 3.84)	0.606	1.53 (0.58, 4.05)	0.388	1.29 (0.40, 4.10)	0.672	2.99 (0.77, 11.63)	0.189

CD, Crohn's disease; UC, ulcerative colitis; IBDU, IBD-unclassified; IC, indeterminate colitis; GI, gastrointestinal tract.

‡. Gastrointestinal phenotypes labeled as IBD without specifying the exact subtype.

§. Other chronic intestinal inflammation resembling IBD, see detailed definitions in Supplementary Table 1B.

¶. The upper gastrointestinal tract includes the esophagus, stomach, and duodenum.

Supplementary Table 5. Multivariate logistic regression analysis of surgical rates of intestinal resection between LO-mIBD and IO-mIBD, adjusted for gastrointestinal complications and genotypes.

	Model 1		Model 2 (adjusted for intestinal fistula)		Model 3 (adjusted for perforation)		Model 4 (adjusted for stricture)		Model 4 (adjusted by genotypes)	
	**OR (95%CI)**	**P value**	**OR (95%CI)**	**P value**	**OR (95%CI)**	**P value**	**OR (95%CI)**	**P value**	**OR (95%CI)**	**P value**
**Intestinal resection**	4.08 (2.42, 6.87)	**<0.001**	4.89 (2.75, 8.68)	**<0.001**	5.13 (2.85, 9.21)	**<0.001**	3.70 (2.07, 6.62)	**<0.001**	3.43 (1.68, 7.00)	**0.001**

Supplementary Table 6. Sensitivity analysis of demographic and clinical characteristics in patients with late-onset and infantile-onset mIBD

	Original analysis			Sensitivity analysis				
	LO-mIBD	IO-mIBD	P value	LO-mIBD		IO-mIBD		P value
	% (N†)	% (N†)		n‡	%	n‡	%	
**Parental consanguinity**	17.4 (92)	30.1 (356)	**0.015**	19	17.3	119	27.3	**0.031**
**Family history of similar monogenic disorders**	45.7 (92)	36.5 (356)	0.108	46	41.8	159	36.5	0.300
**Family history of IBD-like manifestations**	28.3 (92)	31.5 (356)	0.553	31	28.2	125	28.7	0.919
**GI symptoms**								
Abdominal pain	46.2 (93)	15.7 (344)	**<0.001**	47	42.7	68	15.6	**<0.001**
Diarrhea	66.7 (93)	92.4 (344)	**<0.001**	73	66.4	362	83.0	**<0.001**
Hematochezia	31.2 (93)	60.8 (344)	**<0.001**	34	30.9	238	54.6	**<0.001**
Perianal disease	10.6 (94)	53.7 (376)	**<0.001**	12	10.9	218	50.0	**<0.001**
**GI complications**								
Intestinal fistula	2.2 (89)	5.7 (335)	0.294	2	1.8	22	5.0	0.224
Perforation	4.5 (89)	7.5 (335)	0.324	5	4.5	29	6.7	0.414
Stricture	31.5 (89)	17.0 (335)	**0.002**	31	28.2	74	17.0	**0.008**
**Lesion sites**								
Esophagus/Stomach	23.6 (89)	19.0 (343)	0.328	23	20.9	83	19.0	0.657
Duodenum	20.2 (89)	25.1 (343)	0.340	22	20.0	98	22.5	0.575
Small intestine	58.4 (89)	29.4 (343)	**<0.001**	58	52.7	128	29.4	**<0.001**
Colon	62.9 (89)	87.2 (343)	**<0.001**	69	62.7	340	78.0	**0.001**
Rectum	11.2 (89)	22.2 (343)	**0.021**	12	10.9	86	19.7	**0.031**
Upper GI involvement	31.5 (89)	31.2 (343)	0.962	31	28.2	136	31.2	0.540
**Endoscopic findings**								
Ulcer	90.9 (66)	75.7 (210)	**0.008**	80	72.7	330	75.7	0.521
Erosion	18.2 (66)	22.9 (210)	0.422	20	18.2	74	17.0	0.764
Pseudopolyps	4.5 (66)	18.6 (210)	**0.006**	5	4.5	60	13.8	**0.008**
**Pathological features**								
Villous atrophy	5.5 (55)	22.9 (236)	**0.005**	6	5.5	75	17.2	**0.002**
Chronic active enteritis	63.6 (55)	71.2 (236)	0.272	70	63.6	239	54.8	0.095
Apoptosis or epithelial injury	7.3 (55)	13.6 (236)	0.202	8	7.3	46	10.6	0.303
Eosinophil-rich	7.3 (55)	8.1 (236)	1.000	8	7.3	27	6.2	0.679
Lymphocytic	20.0 (55)	9.3 (236)	**0.025**	17	15.5	41	9.4	0.066
Granulomatous	21.8 (55)	10.6 (236)	**0.024**	18	16.4	46	10.6	0.090
**Extraintestinal manifestations**								
> 1 EIMs	49.1 (110)	51.3 (423)	0.680	54	49.1	220	50.5	0.798
> 2 EIMs	29.1 (110)	26.5 (423)	0.582	32	29.1	116	26.6	0.600
Anemia	30.9 (110)	18.4 (423)	**0.004**	34	30.9	80	18.3	**0.004**

	Original analysis			Sensitivity analysis				
	**LO-mIBD**	**IO-mIBD**	**P value**	**LO-mIBD**		**IO-mIBD**		**P value**
	**% (N†)**	**% (N†)**		**n‡**	**%**	**n‡**	**%**	
Growth retardation	11.8 (110)	44.9 (423)	**<0.001**	13	11.8	193	44.3	**<0.001**
Oral lesions	10.0 (110)	22.0 (423)	**0.005**	11	10.0	94	21.6	**0.006**
Skin lesions	29.1 (110)	36.9 (423)	0.128	32	29.1	158	36.2	0.160
Musculoskeletal abnormality	29.1 (110)	10.6 (423)	**<0.001**	32	29.1	46	10.6	**<0.001**
Hepato-splenic-biliary abnormality	13.6 (110)	13.9 (423)	0.933	15	13.6	60	13.8	0.973
Chronic renal disease	1.8 (110)	1.9 (423)	1.000	2	1.8	8	1.8	1.000
Chronic pulmonary disease	7.3 (110)	3.3 (423)	0.111	8	7.3	14	3.2	0.096
Cardiovascular abnormality	3.6 (110)	4.3 (423)	0.983	4	3.6	18	4.1	1.000
Endocrine abnormality	5.5 (110)	4.0 (423)	0.692	6	5.5	17	3.9	0.645
Hematological abnormality	10.9 (110)	7.6 (423)	0.256	12	10.9	33	7.6	0.255
Neurological abnormality	9.1 (110)	5.4 (423)	0.157	10	9.1	24	5.5	0.164
Ocular and auditory abnormality	1.8 (110)	2.8 (423)	0.794	2	1.8	12	2.8	0.829
Lymphadenopathy	7.3 (110)	4.0 (423)	0.150	8	7.3	17	3.9	0.130
**Comorbidities**								
Recurrent infections	30.0 (110)	41.8 (423)	**0.024**	33	30.0	179	41.1	**0.034**
Relapsing fever	8.2 (110)	22.5 (423)	**0.001**	9	8.2	96	22.0	**0.001**
Autoimmune disease	10.9 (110)	9.0 (423)	0.537	12	10.9	39	8.9	0.527
Autoinflammation	8.2 (110)	10.9 (423)	0.408	9	8.2	46	10.6	0.461
HLH/MAS	0.9 (110)	3.3 (423)	0.302	1	0.9	14	3.2	0.320
Allergy	0.9 (110)	2.4 (423)	0.562	1	0.9	10	2.3	0.587
Ectodermal dysplasia	3.6 (110)	5.7 (423)	0.394	4	3.6	24	5.5	0.427
Dysmorphism	3.6 (110)	6.4 (423)	0.273	4	3.6	27	6.2	0.301
Malignancy	5.5 (110)	3.8 (423)	0.606	6	5.5	16	3.7	0.562

†. N = number of cases with available information about the features in the reports.

‡. n = the expected number of cases based on the assumed $RI_{LTFU/FU}$.

Supplementary Table 7. Treatment interventions in patients with late-onset and infantile-onset mIBD

	LO-mIBD		IO-mIBD		P value
	n/N†	%	n/N†	%	
**Conventional Treatment**					
5-ASA	27/90	30.0	108/378	28.6	0.797
GCS	44/90	48.9	218/378	57.7	0.156
IS	21/90	23.3	208/378	55.0	**<0.001**
**Biologics/small molecules**					
Anti-TNFα	22/90	24.4	114/378	30.2	0.304
Anti-IL12/23	4/90	4.4	1/378	0.3	**0.006**
Anti-integrin	3/90	3.3	6/378	1.6	0.384
Anti-JAK/STAT	0/90	0.0	4/378	1.1	1.000
Anti-IL1β/1R	0/90	0.0	17/378	4.5	0.053
Anti-CD20 (Rituximab)	1/90	1.1	2/378	0.5	0.474
Anti-IL6R (Tocilizumab)	0/90	0.0	1/378	0.3	1.000
CTLA4-Ig (Abatacept)	2/90	2.2	2/378	0.5	0.168
**Anti-TNFα plus IS**	7/90	7.8	96/378	25.4	**<0.001**
**Anti-IL12/23 plus IS**	1/90	1.1	1/378	0.3	0.348
**Anti-integrin plus IS**	1/90	1.1	6/378	1.6	1.000
**Others**					
Antibiotics	15/90	16.7	91/378	24.1	0.161
Enteral nutrition	3/90	3.3	31/378	8.2	0.120
Parenteral nutrition	2/90	2.2	104/378	27.5	**<0.001**
IVIG	19/90	21.1	60/378	15.9	0.273
**Surgery**					
Intestinal resection	34/90	37.8	50/378	13.2	**<0.001**
Perianal surgery‡	1/10	10.0	26/194	13.4	1.000
**HSCT**	3/90	3.3	100/378	26.5	**<0.001**

5-ASA, 5-aminosalicylic acid; GCS, Glucocorticoids; IS, immune suppressant and modulators, including azathioprine, methotrexate, cyclosporine, thalidomide, tacrolimus, and sirolimus; TNFα, tumor necrosis factor alpha; IVIG, Intravenous immunoglobulin; HSCT, hematopoietic stem cell transplantation.

†. n = number of cases presenting the corresponding features; N = number of cases with available information about the features in the reports.

‡. Calculated based on cases presenting with perianal lesions.

Supplementary Table 8. Genetic features and mechanisms of the top 14 pathogenic genes in patients with late-onset and infantile-onset mIBD

Gene	Inher-itance	Disease syndrome	Pathways	Mechanisms related to intestinal inflammation	Ref
**LO-mIBD**					
SLCO2A1	AR	primary hypertrophic osteoarthropathy (PHO)	Lipid and prostaglandin transport	Defective prostaglandin transport, mucosal barrier damage	[3, 4]
GUCY2C	AD	Congenital diarrhea	cGMP biosynthesis and guanylyl cyclase (GC) signaling	Disrupted intraluminal electrolyte milleu & dysbiosis	[5, 6]
CTLA4	AD	IPEX (-like)	Treg transcription, CD28 co-stimulation, TCR	dysregulation of Treg and hyperactivation of effector T cells	[7, 8]
SLC26A3	AR	Congenital diarrhea	Bicarbonate and chloride transport	Disrupted intraluminal electrolyte milleu & dysbiosis	[9-11]
TYMP	AR	Mitochondrial neurogastrointestinal encephalomyopathy (MNGIE)	Mitochondrial DNA, apoptosis	Dysmotility and/or linked to the mitochondrial dysfunction of intestinal cells	[12, 13]
SYK	AD	Immunodeficiency and systemic inflammatory disease	AKT, NF-κB, BCR signaling, FCγR-phagocytosis	Hyperinflammatory response to dectin ligands	[14, 15]
ICOS	AR	Common variable immunodeficiency (CVID)	AKT, NF-κB, CD28 signaling	Insufficient IL-10 production by T cells and impaired co-stimulation	[16, 17]
BTK	XLR	X-linked agammaglobulinemia (XLA)	NF-κB, TLR, FCγR-phagocytosis,	Impaired B cell function and heightened Th1 response	[18, 19]
HPS1	AR	Hermansky-pudlak syndrome (HPS)	Vesicle-mediated transport	Impaired antimicrobial activity due to defective vesicle transport	[20, 21]
NCF1	AR	Chronic granulomatous disease (CGD)	ROS production, RHO GTPase	Impaired antimicrobial activity due to NADPH oxidase defect	[22-24]
G6PC3	AR	Congenital neutropenia	AKT, gluconeogenesis, carbohydrate biosynthesis	Impaired neutrophil function and antimicrobial activity	[14, 25, 26]
**Overlapping in LO-mIBD and IO-mIBD**					
CYBB	XLR	Chronic granulomatous disease (CGD)	ROS production, RHO, RAC1 & 2 GTPase	Impaired antimicrobial activity due to NADPH oxidase defect	[22-24]
XIAP	XLR	X-linked lymphoproliferative disease	RIPK1 necrosis, TNFα, apoptosis,	Dysregulation of cell apoptosis and impaired pathogen recognition	[14, 27-29]

Gene	Inher-itance	Disease syndrome	Pathways	Mechanisms related to intestinal inflammation	Ref
		(XLP)	NOD2 signaling		
TNFAIP3	AD	A20 haploinsufficiency (HA20)	NF-KB, TNFα	Increased expression of NF- κB- mediated proinflammatory cytokines	30, 31
**IO-mIBD**					
IL10RA	AR	Infantile enterocolitis and fistulizing disease	IL10 signaling	Impaired IL-10 mediated control of inflammatory responses with IL-1 and IL-23	14, 32-35
IL10RB	AR	Infantile enterocolitis and fistulizing disease	IL10 signaling	Impaired IL-10 mediated control of inflammatory responses with IL-1 and IL-23	14, 32-35
TTC7A	AR	Intestinal atresia and immunodeficiency	Cell cycle, protein transport	Defective epithelial polarization/apoptosis and lymphocyte selection defect	36-39
FOXP3	XLR	IPEX (-like)	Treg transcription	Defective in regulatory T cell differentiation and activity with impaired effector cell control	40-42
RIPK1	AR	Immunodeficiency with autoinflammation	NF-KB and TLR signaling, RIPK1 necrosis, TNFα	Aberrant TNF-induced cell death with epithelial barrier erosion	43-45
LRBA	AR	IPEX (-like)	Mitophagy, protein localization	Defective in regulatory T cell differentiation and activity with impaired effector cell control	46, 47
SKIV2L	AR	Trichohepatoenteric syndrome (THES)	mRNA processing, RIG- I- like receptor (RLR)	Likely dominant epithelial defect in combination with a lymphocyte selection defect	48-50
NLRC4	AD	Macrophage activation syndrome (MAS)-like	NOD2 signaling, inflammasome, apoptosis	NLRC4 inflammasome activation, IL-1 and IL-18 secretion	51, 52
TTC37	AR	Trichohepatoenteric syndrome (THES)	mRNA processing	Likely dominant epithelial defect in combination with a lymphocyte selection defect	53, 54
DKC1	XLR	Dyskeratosis congenita	Telomere extension	T-cell deficiency and defective epithelial barrier function	55-57
MVK	AR	Hyper IgD syndrome (HIDS)	Cholesterol biosynthetic	Hyperinflammation due to defective mevalonate metabolism, inflammasome and IL-1 activation	58-60

Data are referred to the On-line Mendelian Inheritance in Man (OMIM)

AR, autosomal recessive; AD, autosomal dominant; XLR, X-linked recessive.

Supplementary Table 9. Gene ontology (GO) enrichment analysis for the most common pathogenic genes in patients with late-onset and infantile-onset mIBD (top 11 gene defects after excluding 3 overlapping genes, CYBB, XIAP, and TNFAIP3)

LO-mIBD				IO-mIBD			
**GO ID**	**GO Term**	**-Log10(*p*)**	**Gene fraction (%)**	**GOID**	**GO Term**	**-Log10(*p*)**	**Gene fraction (%)**
GO:0071226	Cellular response to molecule of fungal origin	7.17×10^{-7}	45.45	GO:0004920	Interleukin-10 receptor activity	1.20×10^{-7}	36.36
hsa04380	Osteoclast differentiation	1.61×10^{-5}	27.27	GO:0050727	Regulation of inflammatory response	2.49×10^{-7}	54.54
GO:0030139	Endocytic vesicle	2.38×10^{-4}	27.27	GO:0055087	Ski complex	3.59×10^{-7}	36.36
GO:0050863	Regulation of T cell activation	3.20×10^{-4}	27.27	GO:0004496	Mevalonate kinase activity	3.63×10^{-4}	18.18
GO:0015711	Organic anion transport	3.47×10^{-4}	27.27	GO:0070926	Regulation of ATP:ADP antiporter activity	3.63×10^{-4}	27.27
GO:0016170	Interleukin-15 receptor binding	3.63×10^{-4}	18.18	GO:0000495	Box H/ACA sno(s)RNA 3'-end processing	7.25×10^{-4}	18.18
GO:0031085	BLOC-3 complex	7.25×10^{-4}	27.27	GO:0032002	Interleukin-28 receptor complex	1.09×10^{-3}	27.27
GO:0009032	Thymidine phosphorylase activity	7.25×10^{-4}	27.27	GO:0072557	IPAF inflammasome complex	1.45×10^{-3}	27.27
GO:0004346	Glucose-6-phosphatase activity	1.09×10^{-3}	18.18	GO:0034497	Protein localization to phagophore assembly site	5.79×10^{-3}	9.09
GO:0048469	Cell maturation	2.00×10^{-3}	27.27	GO:0046854	Phosphatidylinositol phosphate biosynthetic process	2.37×10^{-2}	18.18
GO:0004383	Guanylate cyclase activity	2.90×10^{-3}	2/11				

GO annotation and enrichment analysis were conducted using Metascape (https://metascape.org/gp/index.html#/).

References

1 Klein C, Lohmann-Hedrich K, Rogaeva E*, et al.* Deciphering the role of heterozygous mutations in genes associated with parkinsonism. *Lancet Neurol*. 2007; 6: 652-62.

2 Wilkins BJ, Kelsen JR, Conrad MA. A pattern-based pathology approach to very early-onset inflammatory bowel disease: thinking beyond Crohn disease and ulcerative colitis. *Adv Anat Pathol*. 2022; 29: 62-70.

3 Rodriguez-Lagunas MJ, Martin-Venegas R, Moreno JJ*, et al.* PGE2 promotes Ca2+-mediated epithelial barrier disruption through EP1 and EP4 receptors in Caco-2 cell monolayers. *Am J Physiol Cell Physiol*. 2010; 299: C324-34.

4 Xie ZX, Li Y, Yang AM*, et al.* Pathogenesis of chronic enteropathy associated with the SLCO2A1 gene: Hypotheses and conundrums. *World J Gastroenterol*. 2024; 30: 2505-11.

5 Fiskerstrand T, Arshad N, Haukanes BI*, et al.* Familial diarrhea syndrome caused by an activating GUCY2C mutation. *N Engl J Med*. 2012; 366: 1586-95.

6 Mishra V, Bose A, Kiran S*, et al.* Gut-associated cGMP mediates colitis and dysbiosis in a mouse model of an activating mutation in GUCY2C. *J Exp Med*. 2021; 218.

7 Kuehn HS, Ouyang W, Lo B*, et al.* Immune dysregulation in human subjects with heterozygous germline mutations in CTLA4. *Science*. 2014; 345: 1623-7.

8 Zeissig S, Petersen BS, Tomczak M*, et al.* Early-onset Crohn's disease and autoimmunity associated with a variant in CTLA-4. *Gut*. 2015; 64: 1889-97.

9 Xiao F, Juric M, Li J*, et al.* Loss of downregulated in adenoma (DRA) impairs mucosal HCO3(-) secretion in murine ileocolonic inflammation. *Inflamm Bowel Dis*. 2012; 18: 101-11.

10 Xiao F, Yu Q, Li J*, et al.* Slc26a3 deficiency is associated with loss of colonic HCO3 (-) secretion, absence of a firm mucus layer and barrier impairment in mice. *Acta Physiol (Oxf)*. 2014; 211: 161-75.

11 Kini A, Singh AK, Riederer B*, et al.* Slc26a3 deletion alters pH-microclimate, mucin biosynthesis, microbiome composition and increases the TNFalpha expression in murine colon. *Acta Physiol (Oxf)*. 2020; 230: e13498.

12 Alcala-Gonzalez LG, Accarino A, Marti R*, et al.* Distinctive gastrointestinal motor dysfunction in patients with MNGIE. *Neurogastroenterol Motil*. 2023; 35: e14643.

13 Kucerova L, Dolina J, Dastych M*, et al.* Mitochondrial neurogastrointestinal encephalomyopathy imitating Crohn's disease: a rare cause of malnutrition. *J Gastrointestin Liver Dis*. 2018; 27: 321-5.

14 Bolton C, Smillie CS, Pandey S, *et al.* An integrated taxonomy for monogenic inflammatory bowel disease. *Gastroenterology*. 2022; 162: 859-76.

15 Wang L, Aschenbrenner D, Zeng Z, *et al.* Gain-of-function variants in SYK cause immune dysregulation and systemic inflammation in humans and mice. *Nat Genet*. 2021; 53: 500-10.

16 Warnatz K, Bossaller L, Salzer U, *et al.* Human ICOS deficiency abrogates the germinal center reaction and provides a monogenic model for common variable immunodeficiency. *Blood*. 2006; 107: 3045-52.

17 Takahashi N, Matsumoto K, Saito H, *et al.* Impaired CD4 and CD8 effector function and decreased memory T cell populations in ICOS-deficient patients. *J Immunol*. 2009; 182: 5515-27.

18 Guan D, Wang Z, Huo J, *et al.* Bruton's tyrosine kinase regulates gut immune homeostasis through attenuating Th1 response. *Cell Death Dis*. 2021; 12: 431.

19 Pal Singh S, Dammeijer F, Hendriks RW. Role of Bruton's tyrosine kinase in B cells and malignancies. *Mol Cancer*. 2018; 17: 57.

20 Yu J, He X, Wei A, *et al.* HPS1 Regulates the Maturation of Large Dense Core Vesicles and Lysozyme Secretion in Paneth Cells. *Front Immunol*. 2020; 11: 560110.

21 Cavounidis A, Pandey S, Capitani M, *et al.* Hermansky-Pudlak syndrome type 1 causes impaired anti-microbial immunity and inflammation due to dysregulated immunometabolism. *Mucosal Immunol*. 2022; 15: 1431-46.

22 Rider NL, Jameson MB, Creech CB. Chronic Granulomatous Disease: Epidemiology, Pathophysiology, and Genetic Basis of Disease. *J Pediatric Infect Dis Soc*. 2018; 7: S2-S5.

23 Marks DJ, Miyagi K, Rahman FZ, *et al.* Inflammatory bowel disease in CGD reproduces the clinicopathological features of Crohn's disease. *Am J Gastroenterol*. 2009; 104: 117-24.

24 Yu HH, Yang YH, Chiang BL. Chronic Granulomatous Disease: a Comprehensive Review. *Clin Rev Allergy Immunol*. 2021; 61: 101-13.

25 Cheung YY, Kim SY, Yiu WH, *et al.* Impaired neutrophil activity and increased susceptibility to bacterial infection in mice lacking glucose-6-phosphatase-beta. *J Clin Invest*. 2007; 117: 784-93.

26 Goenka A, Doherty JA, Al-Farsi T, *et al.* Neutrophil dysfunction triggers inflammatory bowel disease in G6PC3 deficiency. *J Leukoc Biol*. 2021; 109: 1147-54.

27 Aguilar C, Lenoir C, Lambert N, *et al.* Characterization of Crohn disease in X-linked inhibitor of apoptosis-deficient male patients and female symptomatic carriers. *J Allergy Clin Immunol*. 2014; 134:

1131-41 e9.

28 Parackova Z, Milota T, Vrabcova P*, et al.* Novel XIAP mutation causing enhanced spontaneous apoptosis and disturbed NOD2 signalling in a patient with atypical adult-onset Crohn's disease. *Cell Death Dis*. 2020; 11: 430.

29 Jost PJ, Vucic D. Regulation of Cell Death and Immunity by XIAP. *Cold Spring Harb Perspect Biol*. 2020; 12.

30 Zhou Q, Wang H, Schwartz DM*, et al.* Loss-of-function mutations in TNFAIP3 leading to A20 haploinsufficiency cause an early-onset autoinflammatory disease. *Nat Genet*. 2016; 48: 67-73.

31 Vereecke L, Beyaert R, van Loo G. The ubiquitin-editing enzyme A20 (TNFAIP3) is a central regulator of immunopathology. *Trends Immunol*. 2009; 30: 383-91.

32 Glocker EO, Kotlarz D, Boztug K*, et al.* Inflammatory bowel disease and mutations affecting the interleukin-10 receptor. *N Engl J Med*. 2009; 361: 2033-45.

33 Shouval DS, Konnikova L, Griffith AE*, et al.* Enhanced TH17 Responses in Patients with IL10 Receptor Deficiency and Infantile-onset IBD. *Inflamm Bowel Dis*. 2017; 23: 1950-61.

34 Zhu L, Shi T, Zhong C*, et al.* IL-10 and IL-10 Receptor Mutations in Very Early Onset Inflammatory Bowel Disease. *Gastroenterology Res*. 2017; 10: 65-9.

35 Aschenbrenner D, Quaranta M, Banerjee S*, et al.* Deconvolution of monocyte responses in inflammatory bowel disease reveals an IL-1 cytokine network that regulates IL-23 in genetic and acquired IL-10 resistance. *Gut*. 2021; 70: 1023-36.

36 Lawless D, Mistry A, Wood PM*, et al.* Bialellic Mutations in Tetratricopeptide Repeat Domain 7A (TTC7A) Cause Common Variable Immunodeficiency-Like Phenotype with Enteropathy. *J Clin Immunol*. 2017; 37: 617-22.

37 Bigorgne AE, Farin HF, Lemoine R*, et al.* TTC7A mutations disrupt intestinal epithelial apicobasal polarity. *J Clin Invest*. 2014; 124: 328-37.

38 Lemoine R, Pachlopnik-Schmid J, Farin HF*, et al.* Immune deficiency-related enteropathy-lymphocytopenia-alopecia syndrome results from tetratricopeptide repeat domain 7A deficiency. *J Allergy Clin Immunol*. 2014; 134: 1354-64 e6.

39 Avitzur Y, Guo C, Mastropaolo LA*, et al.* Mutations in tetratricopeptide repeat domain 7A result in a severe form of very early onset inflammatory bowel disease. *Gastroenterology*. 2014; 146: 1028-39.

40 Torgerson TR, Ochs HD. Immune dysregulation, polyendocrinopathy, enteropathy, X-linked:

forkhead box protein 3 mutations and lack of regulatory T cells. *J Allergy Clin Immunol*. 2007; 120: 744-50; quiz 51-2.

41 Bamidele AO, Svingen PA, Sagstetter MR*, et al.* Disruption of FOXP3-EZH2 Interaction Represents a Pathobiological Mechanism in Intestinal Inflammation. *Cell Mol Gastroenterol Hepatol*. 2019; 7: 55-71.

42 Barzaghi F, Passerini L, Bacchetta R. Immune dysregulation, polyendocrinopathy, enteropathy, x-linked syndrome: a paradigm of immunodeficiency with autoimmunity. *Front Immunol*. 2012; 3: 211.

43 Li Y, Fuhrer M, Bahrami E*, et al.* Human RIPK1 deficiency causes combined immunodeficiency and inflammatory bowel diseases. *Proc Natl Acad Sci U S A*. 2019; 116: 970-5.

44 Takahashi N, Vereecke L, Bertrand MJ*, et al.* RIPK1 ensures intestinal homeostasis by protecting the epithelium against apoptosis. *Nature*. 2014; 513: 95-9.

45 Zhang J, Jin T, Aksentijevich I*, et al.* RIPK1-Associated Inborn Errors of Innate Immunity. *Front Immunol*. 2021; 12: 676946.

46 Lo B, Zhang K, Lu W*, et al.* AUTOIMMUNE DISEASE. Patients with LRBA deficiency show CTLA4 loss and immune dysregulation responsive to abatacept therapy. *Science*. 2015; 349: 436-40.

47 Sudan R, Fernandes S, Srivastava N*, et al.* LRBA Deficiency Can Lead to Lethal Colitis That Is Diminished by SHIP1 Agonism. *Front Immunol*. 2022; 13: 830961.

48 Yang K, Han J, Gill JG*, et al.* The mammalian SKIV2L RNA exosome is essential for early B cell development. *Sci Immunol*. 2022; 7: eabn2888.

49 Eckard SC, Rice GI, Fabre A*, et al.* The SKIV2L RNA exosome limits activation of the RIG-I-like receptors. *Nat Immunol*. 2014; 15: 839-45.

50 Yang K, Han J, Asada M*, et al.* Cytoplasmic RNA quality control failure engages mTORC1-mediated autoinflammatory disease. *J Clin Invest*. 2022; 132.

51 Steiner A, Reygaerts T, Pontillo A*, et al.* Recessive NLRC4-Autoinflammatory Disease Reveals an Ulcerative Colitis Locus. *J Clin Immunol*. 2022; 42: 325-35.

52 Canna SW, de Jesus AA, Gouni S*, et al.* An activating NLRC4 inflammasome mutation causes autoinflammation with recurrent macrophage activation syndrome. *Nat Genet*. 2014; 46: 1140-6.

53 Lee WI, Huang JL, Chen CC*, et al.* Identifying Mutations of the Tetratricopeptide Repeat Domain 37 (TTC37) Gene in Infants With Intractable Diarrhea and a Comparison of Asian and Non-Asian Phenotype and Genotype: A Global Case-report Study of a Well-Defined Syndrome With

Immunodeficiency. *Medicine (Baltimore)*. 2016; 95: e2918.

54 Hartley JL, Zachos NC, Dawood B*, et al.* Mutations in TTC37 cause trichohepatoenteric syndrome (phenotypic diarrhea of infancy). *Gastroenterology*. 2010; 138: 2388-98, 98 e1-2.

55 Knight SW, Heiss NS, Vulliamy TJ*, et al.* Unexplained aplastic anaemia, immunodeficiency, and cerebellar hypoplasia (Hoyeraal-Hreidarsson syndrome) due to mutations in the dyskeratosis congenita gene, DKC1. *Br J Haematol*. 1999; 107: 335-9.

56 Sznajer Y, Baumann C, David A*, et al.* Further delineation of the congenital form of X-linked dyskeratosis congenita (Hoyeraal-Hreidarsson syndrome). *Eur J Pediatr*. 2003; 162: 863-7.

57 Zeng T, Lv G, Chen X*, et al.* CD8(+) T-cell senescence and skewed lymphocyte subsets in young Dyskeratosis Congenita patients with PARN and DKC1 mutations. *J Clin Lab Anal*. 2020; 34: e23375.

58 Romano M, Arici ZS, Piskin D*, et al.* The 2021 EULAR/American College of Rheumatology points to consider for diagnosis, management and monitoring of the interleukin-1 mediated autoinflammatory diseases: cryopyrin-associated periodic syndromes, tumour necrosis factor receptor-associated periodic syndrome, mevalonate kinase deficiency, and deficiency of the interleukin-1 receptor antagonist. *Ann Rheum Dis*. 2022; 81: 907-21.

59 Pontillo A, Paoluzzi E, Crovella S. The inhibition of mevalonate pathway induces upregulation of NALP3 expression: new insight in the pathogenesis of mevalonate kinase deficiency. *Eur J Hum Genet*. 2010; 18: 844-7.

60 Bader-Meunier B, Martins AL, Charbit-Henrion F*, et al.* Mevalonate Kinase Deficiency: A Cause of Severe Very-Early-Onset Inflammatory Bowel Disease. *Inflamm Bowel Dis*. 2021; 27: 1853-7.
